# LncRNA GOLGA2P10 is induced by PERK/ATF4/CHOP signaling and protects tumor cells from ER stress-induced apoptosis by regulating Bcl-2 family members

**DOI:** 10.1038/s41419-020-2469-1

**Published:** 2020-04-24

**Authors:** Meng-Zhi Wu, Tao Fu, Jin-Xi Chen, Ying-Ying Lin, Jin-E Yang, Shi-Mei Zhuang

**Affiliations:** MOE Key Laboratory of Gene Function and Regulation, School of Life Sciences, Collaborative Innovation Center for Cancer Medicine, Sun Yat-sen University, Xin Gang Xi Road 135#, Guangzhou, 510275 P. R. China

**Keywords:** Apoptosis, Long non-coding RNAs

## Abstract

Elevated endoplasmic reticulum (ER) stress is frequently observed in cancers, whereas sustained ER stress may trigger apoptosis. How cancer cells escape from ER stress-induced apoptosis remain unclear. Here, we found that a pseudogene-derived lncRNA, Golgin A2 pseudogene 10 (GOLGA2P10), was frequently upregulated in HCC tissues and significantly elevated in hepatoma cells treated with ER stress inducers, such as tunicamycin and thapsigargin. Higher GOLGA2P10 level was correlated with shorter recurrence-free survival of HCC patients. Upon ER stress, CHOP directly bound to the promoter of GOLGA2P10 and induced its transcription via the PERK/ATF4/CHOP pathway. Interestingly, the ER stress inducer-stimulated apoptosis was promoted by silencing GOLGA2P10 but was antagonized by overexpressing GOLGA2P10. Both gain- and loss-of-function analyses disclosed that GOLGA2P10 increased BCL-xL protein level, promoted BAD phosphorylation, and conferred tumor cells with resistance to ER stress-induced apoptosis. Moreover, BCL-xL overexpression or BAD knockdown abrogated the apoptosis-promoting effect of GOLGA2P10 silencing. Consistently, the Ser75Ala mutation in BAD, which caused phosphorylation-resistance, further enhanced the promoting effect of BAD in tunicamycin-induced apoptosis. These results suggest that ER stress induces GOLGA2P10 transcription through the PERK/ATF4/CHOP pathway, and upregulation of GOLGA2P10 protects tumor cells from the cytotoxic effect of persistent ER stress in tumor microenvironment by regulating Bcl-2 family members, which highlight GOLGA2P10 as a potential target for anticancer therapy.

## Introduction

Long noncoding RNAs (lncRNAs) belong to a class of non-protein coding transcripts that are longer than 200 nucleotides ^1^. A number of lncRNAs have been shown to play vital roles in different physiological and pathological processes, including tumor development^2–4^.

Perturbations to endoplasmic reticulum (ER) by extracellular and intracellular factors, such as nutrient deprivation, hypoxia, high-fat diet, viral infection, and alternations in redox status as well as Ca^2+^ content, can cause accumulation of unfolded or misfolded proteins, resulting in ER stress and subsequent activation of the “unfolded protein response” (UPR)^5^. In response to ER stress, three UPR sensors, including endoribonuclease inositol-requiring enzyme 1-alpha (IRE1α), protein kinase RNA-like ER kinase (PERK), and activating transcription factor 6 (ATF6), are activated by release of the bound negative regulator GRP78 chaperone protein and then induce the expression of genes that are involved in protein folding, secretion, and quality control, which consequently alleviate ER stress^6–9^. Sustained ER stress may result in long-term activation of the UPR axis and trigger apoptotic cell death. C/EBP-homologous protein (CHOP), the downstream gene of UPR sensors, is a transcription factor that regulates the expression of genes involved in ER stress-induced apoptosis^10,11^.

ER stress has been implicated in various pathological processes, like neurodegenerative diseases, diabetes, metabolic syndromes, and tumor development^12–15^. Persistent ER stress is documented in different types of cancer owing to the hypoxic and oxidative tumor environment^13,16^. Hepatocellular carcinoma (HCC) is one of the most common malignancies worldwide^17^. Elevated ER stress is detected in precancerous conditions that precede HCC development, including hepatitis B virus and hepatitis C virus infections and nonalcoholic steatohepatitis^18–21^. To date, how cancer cells escape from ER stress-induced apoptosis and the causative effect of lncRNAs in ER stress-induced apoptosis remain unexplored. In this study, we found a pseudogene-derived lncRNA, Golgin A2 (GOLGA2) pseudogene 10 (GOLGA2P10), was upregulated in HCCs and upon ER stress. The upregulation of GOLGA2P10 was correlated with shorter recurrence-free survival of HCC patients. Further investigation revealed that CHOP directly bound to the GOLGA2P10 promoter and induced its transcription via the PERK/ATF4/CHOP pathway. Both gain- and loss-of-function analyses revealed that GOLGA2P10 conferred tumor cells with resistance to ER stress-induced apoptosis by increasing the protein level of BCL-xL and promoting phosphorylation of BAD. Our data disclose the novel biological function of a lncRNA in ER stress-induced apoptosis, and highlight GOLGA2P10 as a potential target for anticancer therapy.

## Results

### GOLGA2P10 is upregulated in HCC tissues and upon ER stress

**Fig. 1:**
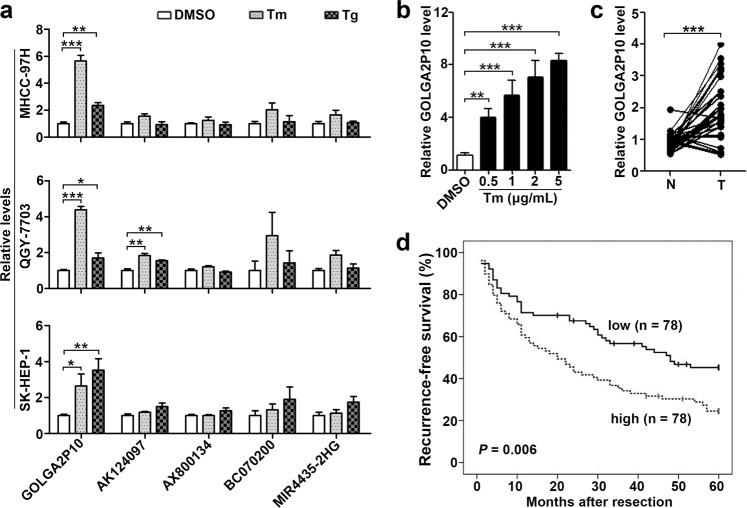
GOLGA2P10 is upregulated in HCC tissues and upon ER stress. **a** Tunicamycin or thapsigargin treatment induced GOLGA2P10 expression. Hepatoma cell lines, including MHCC-97H, QGY-7703, and SK-HEP-1, were treated with DMSO (vehicle control), tunicamycin or thapsigargin for 16 h before qPCR analysis. **b** Tunicamycin treatment increased GOLGA2P10 level in a dose-dependent manner. MHCC-97H cells were treated with DMSO or the indicated doses of tunicamycin for 16 h before qPCR analysis. For **a**, **b**, the mean value of DMSO-treated cells was set as relative level 1. Data are shown as mean ± SEM of three independent experiments. Tm tunicamycin, Tg thapsigargin. **c** GOLGA2P10 was upregulated in HCC tissues. GOLGA2P10 levels were detected in 31 paired HCC (T) and adjacent non-tumor liver tissues (N) by qPCR analysis. The mean value of adjacent non-tumor liver tissues was set as relative level 1. **d** Kaplan–Meier plots revealed a correlation between high GOLGA2P10 expression and short recurrence-free survival. GOLGA2P10 levels were analyzed in HCC tissues (*n* = 156) by qPCR and the median value was chosen as the cut-off point for separating the GOLGA2P10-low level group (*n* = 78) from the GOLGA2P10-high level group (*n* = 78). β-actin was used as an internal control. *P < 0.05; **P < 0.01; ***P < 0.001.

Considering that upregulation of some lncRNAs in tumors may result from persistent ER stress during cancer development and confer tumor cells with survival advantage, we conducted a bioinformatics analysis on the expression profiles (GSE54238) of 10 normal liver and 26 HCC tissues and identified 5 lncRNAs (AK124097, AX800134, BC070200, MIR4435-2HG, and NR_026811) that were located in intergenic regions and upregulated in HCC tissues (Supplementary Fig. 1). To test whether these lncRNAs were upregulated by ER stress, three hepatoma cell lines, including MHCC-97H, QGY-7703, and SK-HEP-1, were treated with ER stress inducer tunicamycin or thapsigargin. As shown, only GOLGA2P10 (NR_026811), a lncRNA transcript of GOLGA2 pseudogene, significantly elevated in all examined cell lines upon treatment of both tunicamycin and thapsigargin (Fig. 1a). Moreover, tunicamycin increased GOLGA2P10 expression in a dose-dependent manner (Fig. 1b). However, other cytotoxic stimuli, like doxorubicin or etoposide, did not affect GOLGA2P10 level (Supplementary Fig. 2a, b). These findings suggest that GOLGA2P10 may be specifically upregulated upon ER stress. Further investigations characterized GOLGA2P10 as a 2872-nt polyadenylated RNA (Supplementary Fig. 3a–c) that was located in both cytoplasmic and nuclear compartments and had no coding ability (Supplementary Fig. 4a–d).

Subsequent analysis showed that 67.7% (21/31) HCCs had more than 50% increases in the GOLGA2P10 level, compared with adjacent non-tumor liver tissues (Fig. 1c). Kaplan–Meier plots revealed a correlation between high GOLGA2P10 expression and short recurrence-free survival (RFS; *P* = 0.006) (Fig. 1d), and multivariate Cox regression analysis further identified high GOLGA2P10 level as an independent risk factor for RFS (hazard ratio = 1.847; *P* = 0.003; Supplementary Table 1). These results indicate GOLGA2P10 as a potential regulator of ER stress-related pathological process.

### ER stress induces GOLGA2P10 transcription via the PERK/ATF4/CHOP pathway

**Fig. 2:**
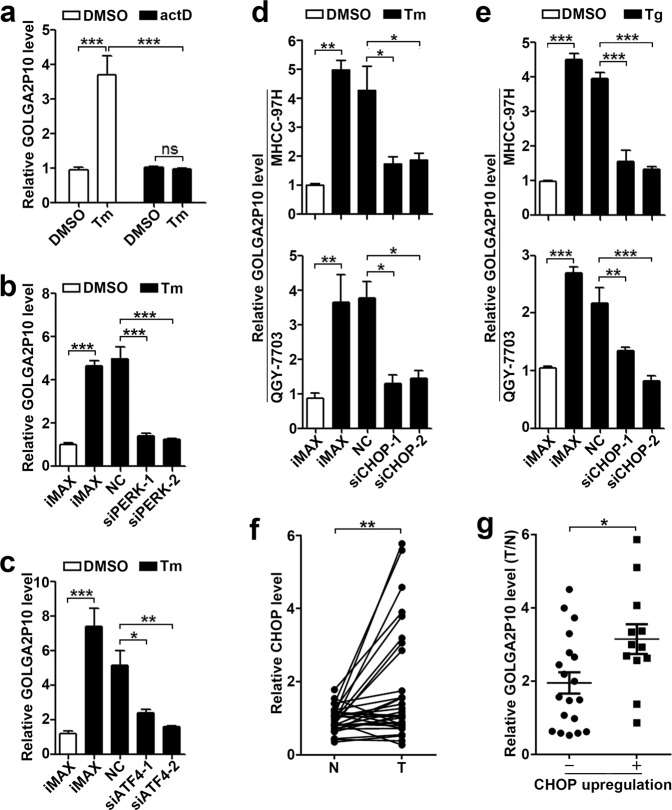
ER stress stimulates GOLGA2P10 transcription via the PERK/ATF4/CHOP signaling pathway. **a** Treatment with actinomycin D abrogated tunicamycin-induced GOLGA2P10 expression. MHCC-97H cells treated with DMSO or tunicamycin were incubated without or with actinomycin D (actD) for 13 h before qPCR analysis. **b**, **c** Silencing of PERK or ATF4 diminished tunicamycin-induced GOLGA2P10 expression in MHCC-97H cells. **d**, **e** CHOP knockdown abrogated tunicamycin- and thapsigargin-induced GOLGA2P10 expression. For **b**–**e**, hepatoma cells were reversely transfected with the indicated RNA duplexes for 36 h, then cultured with DMSO (**b**–**e**), tunicamycin (**b**–**d**), or thapsigargin (**e**) for an additional 16 h before qPCR analysis. iMAX, cells exposed to Lipofectamine RNAiMAX but not RNA duplexes. NC, negative control for siRNAs. For **a**–**e**, data are shown as mean ± SEM of three independent experiments. **f** CHOP was upregulated in HCC tissues. The mRNA level of CHOP was detected in 31 paired HCC (T) and adjacent non-tumor liver tissues (N) by qPCR analysis. The mean value of adjacent non-tumor liver tissues was set as relative level 1. **g** HCCs with CHOP elevation displayed higher GOLGA2P10 expression. The levels of CHOP or GOLGA2P10 in HCC tissue relative to that in adjacent non-tumor tissue (T/N), based on data from Figs. 2f and 1c, were used for analysis. T/N = 1.5 was chosen as the cut-off point for separating the tumors without (−) CHOP-upregulation (*n* = 19) from those with (+) CHOP-upregulation (*n* = 12). β-actin was used as an internal control. *P < 0.05; **P < 0.01; ***P < 0.001; ns not significant.

We next investigated how ER stress induced GOLGA2P10 expression. As shown, the increase of GOLGA2P10 expression resulted from tunicamycin treatment was completely abrogated by transcription inhibitor actinomycin D (Fig. 2a), suggesting that ER stress may stimulate GOLGA2P10 transcription. Furthermore, silencing of PERK, but not IRE1α and ATF6, attenuated tunicamycin-induced GOLGA2P10 expression (Fig. 2b, Supplementary Fig. 5a, b). Consistently, knockdown of either ATF4, the downstream molecule of PERK kinase, or CHOP, the ATF4 target gene, diminished the ER stress-induced GOLGA2P10 expression (Fig. 2c–e, Supplementary Fig. 6a–c). Furthermore, the level of CHOP was increased in 38.7% (12/31) HCC tissues, and among 12 HCCs with CHOP upregulation, 11 displayed enhanced GOLGA2P10 expression (Fig. 2f, g). These findings suggest that ER stress may promote GOLGA2P10 transcription via PERK/ATF4/CHOP pathway.

**Fig. 3:**
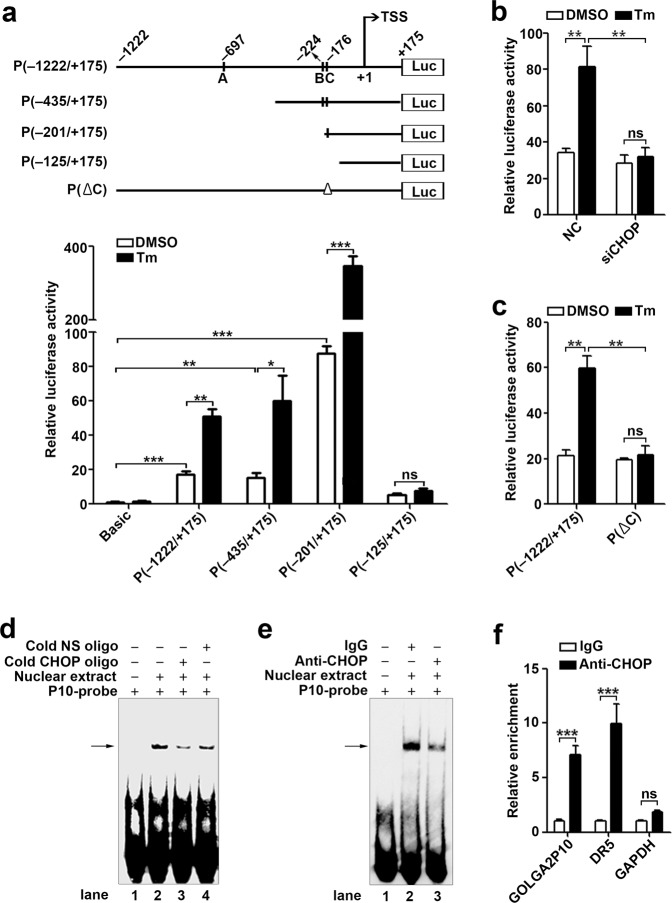
CHOP directly binds to the GOLGA2P10 promoter and transactivates GOLGA2P10 expression under ER stress. **a** Identification of the GOLGA2P10 promoter. Upper panel, schematic diagram of the reporter plasmids containing different fragments of the GOLGA2P10 promoter. Three putative CHOP binding sites, designated as A–C, are shown as short vertical lines. Deletion of site C is depicted as a triangle (∆). TSS transcriptional start site. Bottom panel: luciferase activity assay. Basic, pGL3-basic vector. **b** CHOP knockdown blocked tunicamycin-stimulated luciferase activity of P(−1222/+175). MHCC-97H cells co-transfected with NC or siCHOP, P(−1222/+175) and pRL-TK were treated with DMSO or tunicamycin for 16 h before luciferase assay. **c** Deletion of site C abrogated tunicamycin-stimulated P(−1222/+175) activity. For **a**, **c**, MHCC-97H cells that were co-transfected with the indicated reporters and pRL-TK were treated with DMSO or tunicamycin for 16 hours before luciferase assay. **d** EMSA verified the interaction between nuclear proteins and site C. **e** Antibody-supershift assay identified CHOP as a potential nuclear protein interacting with site C. For **d**, **e**, nuclear extracts were isolated from tunicamycin-treated MHCC-97H cells. Each binding reaction included a biotin-labeled probe that comprised site C sequence (P10-probe). For competition assay, nuclear extracts were preincubated with fivefold molar excess of unlabeled CHOP consensus binding sequence (Cold CHOP oligo) or nonspecific oligonucleotide (Cold NS oligo) prior to adding labeled P10-probe. The arrow indicates the DNA-protein complexes. − absence, + presence. Two independent experiments were performed with similar results. **f** ChIP analysis revealed an in vivo interaction between CHOP and the GOLGA2P10 promoter. MHCC-97H cells were treated with tunicamycin for 8 h before ChIP analysis using antibody against CHOP (anti-CHOP) or isotype-matched IgG. The promoter region of DR5 and GAPDH was used as positive and negative control, respectively. For **a**–**c**, **f**, data are shown as mean ± SEM of three independent experiments. *P < 0.05; **P < 0.01; ***P < 0.001; ns not significant.

Based on chromatin immunoprecipitation (ChIP)-sequencing data, H3K4Me1, H3K4Me3, and H3K27Ac, which represent the histone modification associated with active promoter, were enriched within the 1.2-kb genomic region upstream of the transcriptional start site (assigned as +1) of GOLGA2P10 (Supplementary Fig. 7), and three putative CHOP binding sites (designated as A–C) were predicted within this region (Fig. 3a). We therefore explored whether GOLGA2P10 was a direct transcriptional target of CHOP. Notably, the promoter reporter P(−1222/+175), which contained the −1222 ~ +175-bp genomic sequence of GOLGA2P10, displayed much higher luciferase activity than the control vector pGL3-basic, suggesting that this region may carry GOLGA2P10 promoter (Fig. 3a). Furthermore, the promoter activity of P(−1222/+175) significantly increased upon tunicamycin exposure (Fig. 3a), whereas this stimulatory effect was completely blocked when CHOP was knocked down (Fig. 3b). Subsequent analysis showed that deletion of the 5′-end region containing sites A and B did not reduce the basal and the tunicamycin-stimulated reporter activity, whereas further deletion of the region containing site C dramatically abolished the promoter activity (Fig. 3a). Consistently, individual site C deletion abrogated the tunicamycin-promoted activity of P(−1222/+175) (Fig. 3c). EMSA further revealed that the probe corresponding to site C could form specific complexes with nuclear proteins, as manifested by appearance of a specific band (Fig. 3d, lane 2), and the band intensity of the probe-protein complexes decreased when excess unlabeled oligonucleotide with CHOP consensus binding sequence was added (Fig. 3d, lane 3), but remained unchanged in the presence of a nonspecific scrambled oligonucleotide (Fig. 3d, lane 4). Antibody-supershift assays showed that preincubation with anti-CHOP antibody led to a decrease in the band intensity of the site C probe–protein complexes (Fig. 3e). ChIP assays disclosed that the fragment containing site C and the promoter of positive control gene DR5, but not negative control gene GAPDH, were enriched in the anti-CHOP-precipitated DNA (Fig. 3f). These results indicate a direct interaction between CHOP and site C in vitro and in vivo.

Taken together, upon stimulation of ER stress, CHOP may directly bind to site C in the GOLGA2P10 promoter and transactivate GOLGA2P10 expression.

### GOLGA2P10 confers tumor cells with resistance to ER stress-induced apoptosis by regulating BCL-xL and BAD

**Fig. 4:**
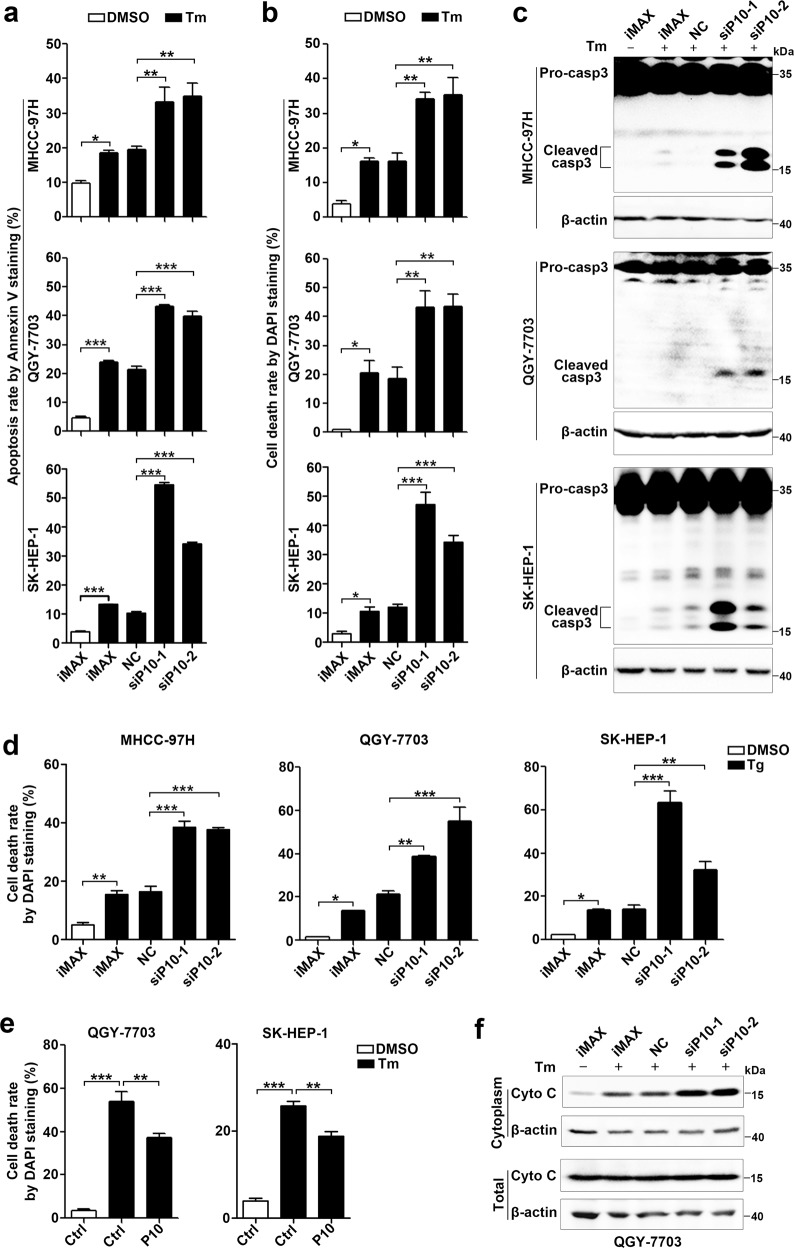
GOLGA2P10 confers tumor cells with resistance to ER stress-induced apoptosis. **a**–**c** Silencing of GOLGA2P10 sensitized hepatoma cells to tunicamycin-induced apoptosis. Tumor cells transfected with the indicated RNA duplexes were treated with DMSO or tunicamycin for 48 (MHCC-97H), 28 (QGY-7703), or 52 h (SK-HEP-1), followed by Annexin V/PI staining and flow cytometry analysis (**a**), DAPI staining and nuclear morphological examination (**b**), or Western blotting analysis for active casepase-3 (**c**). **d** Inhibition of GOLGA2P10 sensitized hepatoma cells to thapsigargin-induced cell death. Hepatoma cells transfected with the indicated RNA duplexes were treated with DMSO or thapsigargin for 48 (MHCC-97H), 28 (QGY-7703), or 52 h (SK-HEP-1), followed by DAPI staining and nuclear morphological examination. **e** Ectopic expression of GOLGA2P10 attenuated tunicamycin-induced cell death. Hepatoma cells with stable overexpression of GOLGA2P10 (P10) or control (Ctrl) vector were treated with DMSO or tunicamycin for 36 (QGY-7703) or 60 h (SK-HEP-1) before DAPI staining. **f** GOLGA2P10 knockdown promoted the release of cytochrome C from mitochondria to the cytosol. QGY-7703 cells transfected with the indicated RNA duplexes were incubated with DMSO (−) or tunicamycin (+) for 28 h, followed by Western blotting analysis for cytochrome C (Cyto C) levels in the cytoplasm and in the whole cell extract. β-actin was used as an internal control. iMAX, cells exposed to Lipofectamine RNAiMAX but not RNA duplexes. NC, negative control for siRNAs. siP10-1 and siP10-2, siRNAs targeting different regions of GOLGA2P10. For **a**, **b**, **d**, **e**, data are shown as mean ± SEM of three independent experiments. For Western blotting in **c**, **f**, two independent experiments were performed with similar results. *P < 0.05; **P < 0.01; ***P < 0.001.

**Fig. 5:**
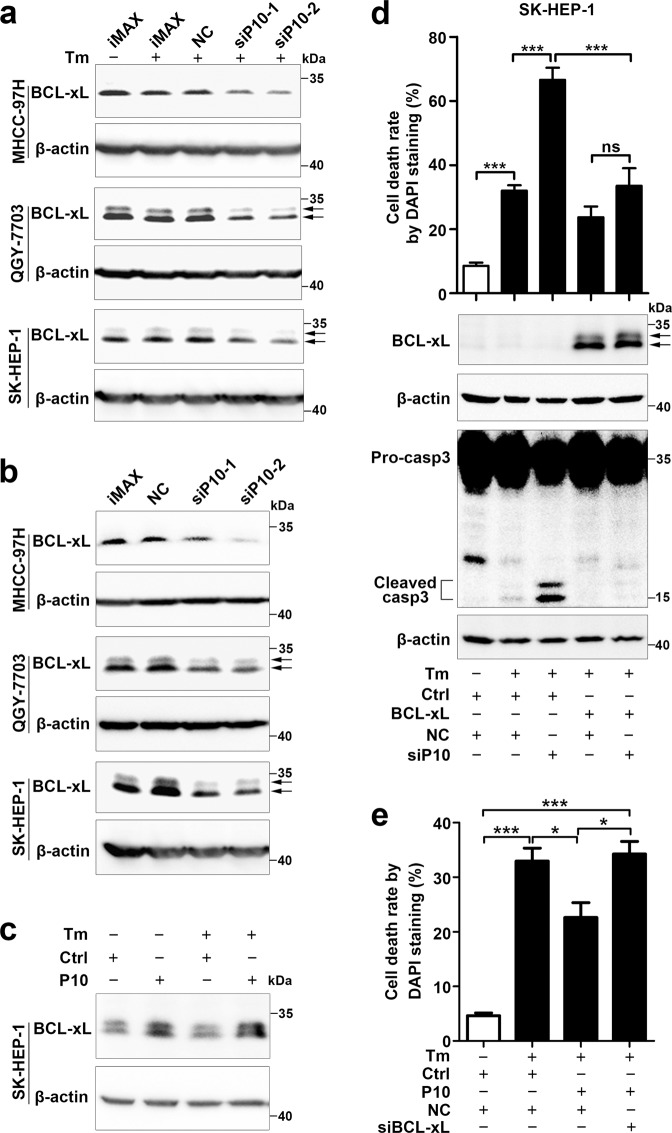
GOLGA2P10 suppresses ER stress-induced apoptosis by increasing BCL-xL level. **a**, **b** GOLGA2P10 knockdown decreased BCL-xL protein level. Hepatoma cells transfected with the indicated RNA duplexes were incubated with DMSO (−) or tunicamycin (+) for 6–8 h (**a**) or without any treatment (**b**), followed by Western blotting. **c** Ectopic expression of GOLGA2P10 increased BCL-xL level. SK-HEP-1 cells with stable overexpression of GOLGA2P10 (P10) or control (Ctrl) vector were treated with DMSO (−) or tunicamycin (+) for 6 h, followed by Western blotting. **d** Ectopic expression of BCL-xL abrogated the pro-apoptosis effect of GOLGA2P10 silencing. SK-HEP-1 cells with stable overexpression of BCL-xL or control (Ctrl) vector were transfected with NC or siP10 for 24 h, then treated with DMSO (−) or tunicamycin (+) for 60 h, followed by DAPI staining or Western blotting. **e** Knockdown of BCL-xL abrogated the anti-apoptosis effect of GOLGA2P10. SK-HEP-1 cells with stable overexpression of GOLGA2P10 (P10) or control (Ctrl) vector were transfected with NC or siBCL-xL for 24 h, then treated with DMSO (−) or tunicamycin (+) for 48 h, followed by DAPI staining. − absence, + presence. β-actin was used as an internal control. For DAPI staining analyses in (**d**, **e**), data are shown as mean ± SEM of three independent experiments. For Western blotting in **a**–**d**, two independent experiments were performed with similar results. *P < 0.05; ***P < 0.001; ns not significant.

We then examined whether GOLGA2P10 expression affected ER stress-induced apoptosis by using Annexin V/propidium iodide (PI) double staining, nuclear morphological examination, and caspase-3 cleavage assays. As shown, silencing GOLGA2P10 (Supplementary Fig. 8) significantly promoted tunicamycin- and thapsigargin-induced apoptosis (Fig. 4a–d, Supplementary Fig. 9), whereas overexpressing GOLGA2P10 antagonized tunicamycin-induced apoptosis (Fig. 4e, Supplementary Fig. 10a, b). Furthermore, inhibition of GOLGA2P10 expression increased the level of cytoplasmic cytochrome C in tunicamycin-treated cells (Fig. 4f), indicating the involvement of mitochondrial apoptosis pathway. We therefore examined the expression of the pro-apoptosis members (BAX, BAK, PUMA, BAD, and BIM) and the anti-apoptosis members (BCL-xL, MCL-1, BCL-2, BCL-w, and BCL2A1) of Bcl-2 family, which are well-known regulators of mitochondrial apoptosis. We found that silencing GOLGA2P10 reduced the BCL-xL protein level, but did not affect its mRNA level, in the cells with or without tunicamycin treatment (Fig. 5a, b, Supplementary Fig. 11). However, GOLGA2P10 knockdown had no effect on the levels of other proteins examined (Supplementary Fig. 12). Consistently, ectopic expression of GOLGA2P10 increased the levels of BCL-xL protein (Fig. 5c) but not other anti-apoptosis molecules of BCL-2 family in the cells without or with tunicamycin treatment (Supplementary Fig. 13). Moreover, the pro-apoptosis effect of GOLGA2P10 silencing was antagonized by BCL-xL overexpression (Fig. 5d), whereas the anti-apoptosis effect of GOLGA2P10 overexpression was abrogated by silencing BCL-xL (Fig. 5e). Subsequent analyses revealed that GOLGA2P10 silencing did not change the RNA levels of the UPR downstream genes, including CHOP, GRP78, GRP94, DR5, GADD34 (Supplementary Fig. 14). These findings suggest that GOLGA2P10 may confer tumor cells with resistance to ER stress-induced apoptosis by enhancing BCL-xL protein level at post-transcriptional level.

**Fig. 6:**
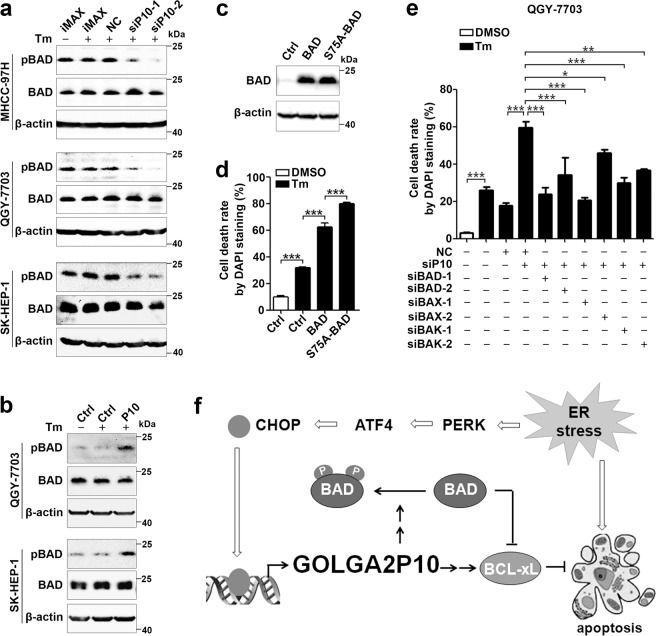
GOLGA2P10 suppresses ER stress-induced apoptosis by enhancing BAD phosphorylation. **a** GOLGA2P10 knockdown inhibited phosphorylation of BAD. Hepatoma cells transfected with the indicated RNA duplexes were incubated with DMSO (−) or tunicamycin (+) for 4 h, followed by Western blotting. pBAD, Ser75-phosphorylated-BAD. **b** Ectopic expression of GOLGA2P10 enhanced phosphorylation of BAD. Hepatoma cells with stable overexpression of GOLGA2P10 (P10) or control (Ctrl) vector were incubated with DMSO (−) or tunicamycin (+) for 4 h before Western blotting. β-actin was used as an internal control. **c**, **d** The promoting effect of BAD in tunicamycin-induced apoptosis was further enhanced when serine 75 of BAD was substituted by alanine. QGY-7703 cells that were transfected with plasmids expressing wild type or Ser75Ala mutant BAD (S75A-BAD) or with control (Ctrl) vector were subjected to Western blotting (**c**), or incubation with DMSO or tunicamycin for 30 h before DAPI staining (**d**). **e** Silencing of BAD, BAX, or BAK abrogated the pro-apoptosis effect of GOLGA2P10 silencing. QGY-7703 cells transfected with the indicated RNA duplexes were treated with DMSO or tunicamycin for 36 h, followed by DAPI staining. − absence, + presence. For DAPI staining analyses in **d**, **e**, data are shown as mean ± SEM of three independent experiments. For Western blotting in **a**–**c**, two independent experiments were performed with similar results. *P < 0.05; **P < 0.01; ***P < 0.001. **f** Schematic diagram of the PERK/ATF**/**CHOP/GOLGA2P10 signaling pathway and its biological function.

It has been shown that BCL-xL inhibits apoptosis by blocking the pro-apoptosis proteins BAK and BAX to form mitochondria permeability transition pores, whereas binding of BAD to BCL-xL suppresses the anti-apoptosis activity of BCL-xL, and this interaction is impaired when BAD is phosphorylated and thereby bound by 14-3-3^22^. As shown, GOLGA2P10 knockdown reduced the level of Ser75-phosphorylated-BAD, but had no effect on the level of total BAD protein (Fig. 6a). On the other hand, GOLGA2P10 overexpression increased phosphorylated-BAD level (Fig. 6b). Moreover, overexpression of BAD promoted tunicamycin-induced apoptosis, and this promoting effect was more pronounced when Ser75Ala-BAD, a phosphorylation-resistant mutant, was transfected (Fig. 6c, d). Consistently, inhibition of BAD, BAX, or BAK expression abolished the apoptosis-promoting effect of GOLGA2P10 silencing in tunicamycin-treated cells (Fig. 6e, Supplementary Fig. 15a–c), suggesting that GOLGA2P10 may repress ER stress-induced apoptosis by enhancing BAD phosphorylation.

Taken together, ER stress may upregulate GOLGA2P10 through the PERK/ATF4/CHOP pathway, and the elevated GOLGA2P10 may confer tumor cells with resistance to ER stress-induced apoptosis by increasing the protein level of BCL-xL and the phosphorylation of BAD (Fig. 6f).

## Discussion

Cancer cells usually grow in hypoxic and oxidative environment, accompanied by prolonged ER stress. It is still unclear how tumor cells evade ER stress-induced apoptosis and survive under harsh conditions, and the causative effect of lncRNA in ER stress regulation remains unexplored. In this study, we identify a new lncRNA GOLGA2P10 and reveal that it is induced by ER stress and prevents cancer cells from ER stress-induced apoptosis by upregulating BCL-xL and increasing BAD phosphorylation, indicating GOLGA2P10 as a potential target for cancer therapy.

Our data suggest that ER stress induces GOLGA2P10 expression via the PERK/ATF4/CHOP pathway, based on following evidences: (1) ER inducers tunicamycin and thapsigargin increase GOLGA2P10 level in multiple tumor cell lines. (2) Knockdown of UPR sensor PERK and its downstream factors, ATF4 and CHOP, suppresses ER stress-induced GOLGA2P10 expression. (3) Deletion of the CHOP binding site C in the GOLGA2P10 promoter blocks tunicamycin-induced promoter activity. (4) EMSA, antibody supershift and ChIP assays show that CHOP directly interacts with the promoter of GOLGA2P10. (5) The levels of both CHOP and GOLGA2P10 are significantly increased in HCC tissues, and HCCs with CHOP upregulation display enhanced GOLGA2P10 expression. To be note, deletion of –435 ~ –200 sequence results in a dramatic increase in the basal activity of GOLGA2P10 promoter, but does not affect the stimulatory role of tunicamycin in the GOLGA2P10 reporter activity, indicating the existence of ER stress-unrelated transcription suppressive elements within this region.

It has been shown that ER stress acts as a barrier to malignant growth by triggering UPR-dependent apoptosis^23^, largely through CHOP, a downstream effector of the PERK/ATF4 signaling pathway. In response to ER stress, CHOP is induced and then translocates into the nucleus, where it regulates gene expression by forming hetero-dimers with other transcription factors. CHOP has been shown to regulate apoptosis by upregulating a number of pro-apoptosis molecules, including BIM^24^, DR5^25^, PUMA^26^, and GADD34^27^, or by downregulating the anti-apoptosis protein BCL-2^28^. Interestingly, it has been shown that CHOP is also activated in various types of human tumors^29–31^. High CHOP level is detected in high-fat diet-induced spontaneous HCCs in *MUP-uPA* mice^32^ and in DEN-induced HCCs^29^. It remains unexplored how tumor cells overcome the pro-apoptosis effect imposed by CHOP. Our results suggest that GOLGA2P10, whose expression is induced by CHOP, may work as a feedback to balance the effect of CHOP-induced pro-apoptosis molecules and thereby prevent CHOP-stimulated apoptosis. Cancer cells with abnormal upregulation of GOLGA2P10 may gain survival advantage. As reported, BAD forms heterodimers with anti-apoptosis proteins BCL-xL or BCL-2 at the outer mitochondrial membrane and represses their death repressor activity, whereas phosphorylated BAD is bound and sequestered by 14-3-3 protein, which hampers formation of BAD/BCL-xL or BAD/BCL-2 dimers^33–35^. We reveal that GOLGA2P10 prevented ER stress-triggered apoptosis by simultaneously increasing protein level of BCL-xL and promoting phosphorylation of BAD. These findings provide new insights into the mechanism underlying the resistance of tumor cells to ER-stress-induced apoptosis and suggest that remodeling the regulatory networks of CHOP-associated cell death may represent a strategy for cancer cells to gain survival advantage.

We show that GOLGA2P10 increases the level of BCL-xL protein but not its mRNA level, suggesting that GOLGA2P10 may regulate BCL-xL post-transcriptionally. Recently, a new functional class of antisense lncRNAs, designated as short interspersed nuclear element-containing translation up-regulators (SINEUPs), was reported to enhance the translation of target mRNAs^36^*.* The activity of SINEUPs depends on the base-pairing between SINEUP and target RNA for target specificity, and the SINE repeat element for translation enhancement^37,38^. We performed bioinformatics analysis and found that there is no SINE element in GOLGA2P10 and no complementary base-pairing between GOLGA2P10 and BCL-xL mRNA, indicating that GOLGA2P10 is unlikely to enhance BCL-xL protein level by acting as a SINEUP.

GOLGA2P10 has been annotated as a pseudogene of GOLGA2. Golgin encoded by GOLGA2 is localized to the Golgi and is important for the stacking of Golgi cisternae and vesicular transport^39–41^. Previous studies showed that depletion of GOLGA2 disrupted the ER-to-Golgi trafficking^42^, which may result in primary cargo accumulation in the ER and therefore lead to ER stress. However, we found that neither ER-stress stimuli, like tunicamycin and thapsigargin, nor silencing GOLGA2P10 changed the protein level of GOLGA2 (Supplementary Fig. 16a, b), inferring that GOLGA2 may not be involved in the GOLGA2P10-mediated resistance to ER stress-induced apoptosis.

In conclusion, our study suggests that under ER stress, CHOP activates GOLGA2P10 expression, whereas GOLGA2P10 in turn protects tumor cells from the cytotoxic effect of ER stress by increasing BCL-xL level and enhancing BAD phosphorylation. Upregulation of GOLGA2P10 may confer cancer cells with survival advantage. These findings highlight the importance of a novel identified lncRNA in the regulation of ER stress, disclose new mechanisms of tumor development, and suggest GOLGA2P10 as a potential anticancer target.

## Materials and methods

### Reagents

The following reagents were used: Dimethyl sulfoxide (DMSO, D2650, Sigma-Aldrich, Saint Louis, MO, USA), tunicamycin (A611129, Sangon Biotech, Shanghai, China), thapsigargin (T7458, Life Technologies, Carlsbad, CA, USA). Unless otherwise indicated, a final concentration of 2 μg/ml tunicamycin or thapsigargin was used to treat hepatoma cells. DMSO was used as a vehicle control.

### Cell lines and human tissue specimens

The human hepatoma cell lines MHCC-97H (cat. SCSP-528) and QGY-7703 (cat. TCHu 43) were obtained from Cell Bank of Chinese Academy of Sciences (Shanghai, China). The human hepatoma cell line SK-HEP-1 (ATCC HTB 52™) and embryonic kidney cell line 293T (ATCC CRL-3216^™^) were from American Tissue Culture Colection (ATCC). All cells were cultured in Dulbecco’s modified Eagle’s medium (DMEM; Life Technologies) supplemented with 10% fetal bovine serum (HyClone, Logan, UT, USA). The cell lines with stable overexpression of GOLGA2P10 and BCL-xL were established as described in the Supplementary Materials and Methods.

HCC and adjacent non-tumor liver tissues were collected from patients who underwent radical tumor resection at Sun Yat-sen University Cancer Center in Guangzhou, P.R. China. Both tumor and non-tumor tissues were histologically confirmed. No local or systemic treatment had been conducted before the surgery. After surgical resection, no other anticancer therapy was administered before relapse. Informed consent was obtained from each patient, and the protocol was approved by the Institutional Research Ethics Committee. Tissues were immediately snap frozen in liquid nitrogen until use. The relevant characteristics of the studied subjects are summarized in Supplementary Table 1.

### Oligonucleotides and plasmids

All small interfering RNA (siRNA) duplexes were purchased from RIBOBIO (Guangzhou, China). Small interference RNAs (siRNAs) targeting human ATF4 (NM_001675), ATF6 (NM_007348), BAX (NM_001291428), BAK (NM_001188), BCL-xL (NM_138578), CHOP (NM_001195057), IRE1α (NM_001433), GOLGA2P10 (NR_026811), and PERK (NM_004836) are designed using the online tool siDESIGN (Dharmacon, IL, USA) and designated as siATF4, siATF6, siBAX, siBAK, siBCL-xL, siCHOP, siIRE1α, siP10, and siPERK, respectively. The negative control RNA duplex (NC) for siRNAs is non-homologous to any human genome sequence.

The firefly luciferase reporter vectors P(−1222/+175), P(−435/+175), P(−201/+175), P(−125/+175), and P(∆C) for GOLGA2P10 promoter activity assays, the ORF–GFP fusion protein expression vectors ORF–GFP, GAPDH–GFP and del-ATG–GFP for evaluating the coding ability of GOLGA2P10, the wild type or Ser75Ala mutant BAD expression constructs, and the lentiviral vectors for expressing human GOLGA2P10 and BCL-xL were constructed as described in Supplementary Materials and Methods. The sequences of siRNAs and primers for vector construction are listed in Supplementary Table 2.

### Cell transfection

Transfection of RNA oligonucleotides was performed using Lipofectamine RNAiMAX (Invitrogen, Carlsbad, CA, USA). Unless otherwise indicated, a final concentration of 20 nM of RNA duplex was used. Transfection of plasmids was performed with Lipofectamine 2000 (Invitrogen).

### Analysis of gene expression

Gene expression was analyzed by real-time quantitative polymerase chain reaction (qPCR), Northern blotting, or Western blotting as described in Supplementary Materials and Methods.

### Rapid amplification of cDNA ends (RACE)

The 5′- and 3′-RACE were performed to identify the 5′- and 3′-ends of GOLGA2P10 from adjacent non-tumor liver tissues using the SMARTer™ PCR cDNA Synthesis Kit (TaKaRa, Kyoto, Japan) and 5′ or 3′ RACE system Kit (Invitrogen) as described previously^43^.

### Luciferase reporter assay

MHCC-97H cells grew in a 48-well plate were co-transfected with 100 ng of pRL-TK (Promega, Madison, WI, USA) that expresses *Renilla* luciferase and 200 ng of P(−1222/+175), P(−435/+175), P(−201/+175), P(−125/+175), or P(∆C) together with or without the indicated RNA duplexes for 24 h, then treated with 2 μg/ml tunicamycin or vehicle control (DMSO) for 16 h before luciferase activity was measured using the Dual-Luciferase Reporter Assay System (Promega). Firefly luciferase activity in each sample was normalized to the activity of *Renilla* luciferase.

### Electrophoretic mobility shift assay (EMSA)

EMSA and antibody-supershift assays were conducted as described previously^44^. Briefly, the biotin-labeled probes were incubated with nuclear extracts of MHCC-97H cells at room temperature for 30 min and subjected to native-PAGE. For competition assay, nuclear extract was preincubated with fivefold molar excess of unlabeled oligonucleotides prior to addition of labeled probe. For antibody-supershift assay, nuclear extract was preincubated with anti-CHOP antibody or isotype-matched IgG before adding to the binding reaction solution that contained labeled probe. Detection of biotinylated RNA in blots was performed using Chemiluminescent EMSA Kit (Beyotime, Shanghai, China). The sequences of probes are listed in Supplementary Table 2.

### Chromatin immunoprecipitation (ChIP) assay

Cells were treated with 2 μg/mL tunicamycin for 8 h, and then cross-linked by formaldehyde. The chromatin complexes were immunoprecipitated using anti-CHOP antibody (#ab11419, Abcam, Cambridge, UK) or isotype-matched IgG (used as a negative control), then collected with Protein A/G MagBeads (Bimake, Houston, TX, USA). The immunoprecipitated DNAs were analyzed by qPCR using primers listed in Supplementary Table 2.

### Analysis of cell apoptosis

Apoptosis was assessed using Annexin V/PI double staining, nuclear morphological examination with 4′,6-diamidino-2-phenylindole (DAPI) staining, and caspase-3 cleavage assays. More details are provided in Supplementary Materials and Methods.

### Statistical analysis

The differences of gene expression between the paired HCC and adjacent non-tumor liver tissues were compared by paired Student’s *t* test. Recurrence-free survival was calculated from the date of HCC resection to the time of first recurrence. Patients who were lost to follow-up or who died from causes unrelated to HCC were treated as censored events. Kaplan–Meier plots and Cox proportional hazard regression analyses, which were applied to identify the prognostic factors, were performed using SPSS version 16.0 (SPSS Inc., Chicago, IL, USA). Significant prognostic factors found in the univariate analysis were further evaluated by a multivariate Cox regression analysis.

Data are presented as the mean ± standard error of the mean from at least three independent experiments. The differences between groups were analyzed by unpaired Student’s *t* test when two groups were compared or by one-way or two-way ANOVA when more than two groups were compared. The variances are similar between the groups that are being statistically compared. The statistical tests are justified as appropriate and meet the assumptions of the tests. *P* < 0.05 was considered as statistically significant. All statistical tests were two-sided and performed using GraphPad Prism version 5.0 software (GraphPad Software, Inc., San Diego, CA, USA).

## Supplementary information

sup figure legends and tables

Figure S1

Figure S2

Figure S3

Figure S4

Figure S5

Figure S6

Figure S7

Figure S8

Figure S9

Figure S10

Figure S11

Figure S12

Figure S13

Figure S14

Figure S15

Figure S16
