# Supplementary material for: LncRNA GOLGA2P10 is induced by PERK/ATF4/CHOP signaling and protects tumor cells from ER stress-induced apoptosis by regulating Bcl-2 family members
Source: Cell Death Dis. 2020 Apr 24;11(4):276. doi: 10.1038/s41419-020-2469-1 (PMC7181651; doi:10.1038/s41419-020-2469-1)
Supplement: Supplementary file 1 — sup figure legends and tables [file 41419_2020_2469_MOESM1_ESM.doc]

**LncRNA GOLGA2P10 is induced by PERK/ATF4/CHOP signaling and protects tumor cells from ER stress-induced apoptosis by regulating Bcl-2 family members**

Meng-Zhi Wu, Tao Fu, Jin-Xi Chen, Ying-Ying Lin, Jin-E Yang, Shi-Mei Zhuang

**Supplementary Information**

**1. Supplementary Materials and Methods Page 2-5**

**2. Supplementary Figures Page 6-11**

**3. Supplementary Tables Page 12-17**

**Supplementary Materials and Methods**

**Plasmid construction**

The following plasmids were constructed: firefly luciferase reporters (P(−1222/+175), P(−435/+175), P(−201/+175), P(−125/+175), and P(∆C)); lentivirus expression vectors (pCDH-GOLGA2P10 and pCDH-BCL-xL); GFP-fusion protein expression vectors (ORF-GFP, GAPDH-GFP, del-ATG-GFP), wild type and Ser75Ala mutant BAD (S75A-BAD) expression vectors.

To identify the promoter of GOLGA2P10, firefly luciferase reporter P(−1222/+175) was generated by cloning the potential promoter region that contained the −1222 ~ +175-bp sequence of GOLGA2P10 (chr15:85777651 ~ 85779047) into the *Xho*I/*Hind*Ⅲ sites upstream of the firefly luciferase gene in a pGL3-basic vector (Promega, Madison, WI, USA). The various deletion constructs that removed potential CHOP binding sites or P(∆C) with deleted CHOP binding sequence at site C were generated by fusion PCR based on P(−1222/+175).

To create pCDH-GOLGA2P10, full-length GOLGA2P10 (2872 bp) was inserted into the *Nhe*I/*Eco*RI sites of the lentivirus expression vector pCDH-SV40-polyA, which was generated by cloning SV40-polyA sequence into the *Not*I site in pCDH-CMV-MCS-EF1-copGFP，which carry copGFP expression cassette (System Biosciences, Palo Alto, CA, USA). To construct pCDH-BCL-xL, the coding sequence of BCL-xL was cloned into the *Nhe*I/*Eco*RI sites of pCDH-CMV-MCS-EF1-copGFP vector (System Biosciences).

The pcDNA3.0-based expression vectors of GFP-fusion protein were generated to evaluate the coding ability of GOLGA2P10. To construct the ORF-GFP plasmid, the predicted ORF containing the 5’-UTR of GOLGA2P10 was fused in frame to the N-terminus of GFP coding sequence (without ATG) and cloning into the *Eco*RI/*Xho*I sites of pcDNA3.0 (Invitrogen, Carlsbad, CA, USA). The GAPDH-GFP fusion expression plasmid was used as a positive control, and the del-ATG-GFP plasmid containing GFP expression cassette without ATG as negative control.

To generate BAD expression vectors, the coding sequences of wild type or Ser75Ala mutant BAD were respectively cloned into the *Hind*III/*Eco*RI sites of plasmid pc3-gab, which was previously produced based on pcDNA3.0 (Invitrogen) by replacing the neomycin ORF with an expression cassette of EGFP [^1^](#_ENREF_1).

All primer sequences used for cloning are listed in Supplementary Table 2. All constructs were confirmed by direct sequencing.

**Lentivirus production**

For lentivirus production, HEK293T cells were co-transfected with 1.6 μg of lentivirus expression vector, 1.2 μg of psPAX2 (12260, Addgene, Watertown, MA, USA) and 0.4 μg of pMD.2G (12259, Addgene) in a 6-cm dish for 12 hours, followed by replacement with 4 ml fresh medium. Forty-eight hours later, the lentiviral supernatant was harvested and centrifuged at 800 g for 10 minutes to remove cellular debris, then stored in aliquots at -80 °C until used for cell infection.

**Construction of stable cell lines**

The cell lines stably expressing GOLGA2P10 or BCL-xL were established by infecting SK-HEP-1 or QGY-7703 cells with lentiviruses that expressed human GOLGA2P10 or BCL-xL.

**Analysis of gene expression**

Real-time quantitative polymerase chain reaction (qPCR) was used to detect RNA levels. Nuclear and cytoplasmic fractions were separated using NE-PER Nuclear and Cytoplasmic Extraction Reagent kit (Thermo Scientific, Rockford, IL, USA). Total RNA, nuclear or cytoplasmic RNA were extracted using RNA TRIzol™ reagent (Invitrogen) and reverse-transcribed using M-MLV reverse transcriptase (M1701, Promega). qPCR was performed on a LightCycler 480 (Roche Diagnostics, Germany) using 2×SYBR Green qPCR Master Mix (B21202, Bimake, Houston, TX，USA). All reactions were performed in duplicate. The cycle threshold (Ct) values should differ by less than 0.5 between duplicate wells. The relative expression levels of the target genes were normalized to that of β-actin, which served as an internal control. A 2^-ΔCt^ value was yielded.

The primers used for qPCR are listed in Supplementary Table 2. The amplification efficiency of primers used for qPCR experiments and the Tm of the amplicon calculated by the dissociation curve are listed in Supplementary Table 3.

Northern blotting was used to detect GOLGA2P10 level in stable cell lines. Briefly, RNA was separated on a 1.2% denaturing agarose/formaldehyde gel. Sequences of the probes, which were 5’-end-labeled with biotin, are listed in Supplementary Table 2. The signal was developed with Chemiluminescent Nucleic Acid Detection Module Kit (Thermo Fisher Scientific, Waltham, MA, USA).

Western blotting was performed to determine the protein levels. Briefly, total cell lysates or cytoplasmic lysates were separated in SDS-polyacrylamide gel, transferred to PVDF membrane (Roche Diagnostics, Germany), and then incubated sequentially with primary and secondary antibodies. The signal was developed with ECL kit (ThermoFisher Scientific). The antibodies used included: rabbit monoclonal antibodies against GOLGA2 (cat. 12480), BCL-2 (cat. 4223), BCL-xL (cat.2764), MCL-1 (cat.5453), S75-phosphorylated BAD (cat.5284), BAD (cat.9239), BAX (cat.5023), BAK (cat.12105), BIM (cat.2933), PUMA (cat.12450) and caspase-3 (Cat.9662) from Cell Signaling Technology (CST, Beverly, MA, USA); rabbit polyclonal antibody against BCL-w (D163267) and BCL2A1 (D220158) from Sangon Biotech (Shanghai, China); mouse monoclonal antibodies against cytochrome C (ab110325, Abcam, Cambridge, MA, USA) and β-actin (BM0627, Boster, Wuhan, China).

**Analysis of cell apoptosis**

Apoptosis was evaluated by Annexin V and propidium iodide (PI) double staining, nuclear morphological examination and caspase-3 cleavage assays. The Annexin V/PI assay was conducted using an Annexin V-FITC/PI Apoptosis Detection Kit (Bimake). Cells were harvested and stained with Annexin V-FITC and PI at room temperature for 15 minutes, and then analyzed by flow cytometry (Gallios, Beckman Coulter, Carlsbad，CA, USA), Annexin V positive cells are considered as apoptotic cells. For nuclear morphological examination, cells were stained with 4′,6-diamidino-2-phenylindole dihydrochloride hydrate (DAPI; Sigma-Aldrich, St. Louis, MO，USA) and observed under fluorescence microscopy, those with fragmented or condensed nuclei were considered as apoptotic cells. At least 400 cells were examined for each sample. Caspase-3 was detected by Western blotting using rabbit polyclonal antibody against caspase-3 for both active caspase-3 (17/19 kDa) and pro-caspase-3 (35 kDa).

**Reference**

1 Su, *H. et a*l. MicroRNA-101, down-regulated in hepatocellular carcinoma, promotes apoptosis and suppresses tumorigenicity*. Cancer R*e**s** 69, 1135-1142, (2009).

**Supplementary Figure Legends**

**Supplementary Fig. 1 Screening procedure to identify ER stress-related lncRNAs in HCC.** To identify the candidate lncRNAs, a bioinformatic analysis was conducted based on published lncRNA expression profiles (GSE54238) of 10 normal liver (NL) and 26 HCC tissues. Using a standardized density of 1500 or higher in the array data and a fold change (FC, average gene expression in HCCs relative to that in NLs) of greater than 3 (*P* < 0.05) as cutoff value, we identified 16 lncRNAs that were significantly upregulated in HCCs. Among them, 11 lncRNAs were located within protein-coding genes, the rest 5 intergenic lncRNAs (AK124097, AX800134, BC070200, MIR4435-2HG and NR_026811) were then selected for subsequent analysis, and only NR_026811 (GOLGA2P10) significantly increased upon tunicamycin (Tm) or thapsigargin (Tg) treatment.

**Supplementary Fig. 2 Doxorubicin and etoposide treatment do not affect GOLGA2P10 level.** SK-HEP-1 cells were treated with vehicle control (Ctrl) or 0.25 μM doxorubicin (a) or 100 μM etoposide (b) for 12 hours, followed by qPCR analysis. Dox, doxorubicin. β-actin was used as an internal control. The mean value of the untreated cells was set as relative level 1. Data are shown as mean ± SEM of three independent experiments. ***, *P* < 0.001. ns, not significant.

**Supplementary Fig. 3** **Identification of the full-length GOLGA2P10 transcript. a-b** Identification of the 5’- and 3’-ends of GOLGA2P10. One microgram of RNA from adjacent non-tumor liver tissue was reversely transcribed into cDNA using random primer (6 mer) and SMARTer II A Oligonucleotide for 5’-RACE or 3’SMART CDS Primer II A for 3’-RACE, followed by 5’-RACE (a) or 3’-RACE (b) analysis and sequencing of RACE products. **c** Structure of human GOLGA2P10 gene and the sequence of GOLGA2P10 transcript. Five exons are indicated by different colours.

**Supplementary Fig. 4 GOLGA2P10 is located in both cytoplasm and nucleus, and has no protein-coding potential. a** GOLGA2P10 is located in both cytoplasm and nucleus. The cytoplasm and nucleus of QGY-7703 cells were fractionated and then subjected to RNA isolation and qPCR analysis. β-actin and MALAT1, which are mainly located in cytoplasm and nucleus respectively, were used as controls. Data are shown as mean ± SEM of three independent experiments. **b** The protein-coding potential of GOLGA2P10 was predicted by the CPAT website (http:// lilab.research.bcm.edu/cpat/). **c** Schematic diagram of the expression vectors used to assess the protein-coding potential. The predicted ORF of GOLGA2P10, together with its 5’UTR, was fused in frame to the the N-terminus of GFP coding sequence (without ATG). The GAPDH-GFP fusion plasmid was used as a positive control and del-ATG-GFP plasmid that contains GFP without ATG as a negative control. **d** No coding capability of the predicted ORF of GOLGA2P10 was found. 293T cells were transfected with the indicated plasmids for 48 hours, then analyzed under bright-field and fluorescence microscopy. Cells showed green indicate expression of GFP fusion protein. Two independent experiments were performed with similar results. Scale bar, 25 μm.

**Supplementary Fig. 5** **Effects of silencing UPR sensor genes on tunicamycin-induced GOLGA2P10 expression. a** The knockdown effect of siRNA targeting PERK, IRE1 or ATF6. **b** Knockdown of IRE1α or ATF6 did not affect tunicamycin-induced GOLGA2P10 expression. MHCC-97H cells were reversely transfected with the indicated RNA duplexes for 36 hours, then treated with DMSO (vehicle control) or tunicamycin for 16 hours, followed by qPCR analysis. Tm, tunicamycin. Tg, thapsigargin. iMAX, cells exposed to Lipofectamine RNAiMAX but not RNA duplexes. NC, negative control for siRNAs. β-actin was used as an internal control. Data are shown as mean ± SEM of three independent experiments. *, *P* < 0.05; **, *P* < 0.01; ***, *P* < 0.001; ns, not significant.

**Supplementary Fig. 6** **Knockdown effect of siRNAs targeting ATF4 or CHOP.** MHCC-97H (a, b) or QGY-7703 (c) cells were reversely transfected with the indicated RNA duplexes for 36 hours, then treated with DMSO or tunicamycin for 16 hours, followed by qPCR analysis. iMAX, cells exposed to Lipofectamine RNAiMAX but not RNA duplexes. NC, negative control for siRNAs. β-actin was used as an internal control. Data are shown as mean ± SEM of three independent experiments. **, *P* < 0.01; ***, *P* < 0.001.

**Supplementary Fig. 7** **The features of the GOLGA2P10 promoter.** ChIP-seq profiles for H3K4Me1, H3K4Me3 and H3K27Ac are visualized using UCSC genome browser (http://genome.ucsc.edu/). The transcription direction and the potential promoter region of GOLGA2P10 are indicated. TSS, transcriptional start site, designated as +1.

**Supplementary Fig. 8** **Knockdown effect of siRNA targeting** **GOLGA2P10.** Hepatoma cells were reversely transfected with the indicated RNA duplexes for 48 hours before qPCR analysis. siP10-1 and siP10-2, siRNA targeting different regions of GOLGA2P10. iMAX, cells exposed to Lipofectamine RNAiMAX but not RNA duplexes. NC, negative control for siRNAs. β-actin was used as an internal control. Data are shown as mean ± SEM of three independent experiments. **, *P* < 0.01; ***, *P* < 0.001.

**Supplementary Fig. 9** **GOLGA2P10 knockdown sensitizes tumor cells to tunicamycin-induced apoptosis.** Hepatoma cells were reversely transfected with the indicated RNA duplexes for 24 hours, then incubated with DMSO (−) or tunicamycin (+) for 48 (MHCC-97H), or 28 (QGY-7703), or 52 hours (SK-HEP-1), followed by Annexin V/PI staining and flow cytometry. iMAX, cells exposed to Lipofectamine RNAiMAX but not RNA duplexes. NC, negative control for siRNAs. Three independent experiments were performed with similar results.

**Supplementary Fig. 10** **Hepatoma cell lines with stable expression of GOLGA2P10.** Hepatoma cells were infected with GOLGA2P10-expressing lentivirus (P10) or its control (Ctrl) and then collected at passage 2 or 3, followed by qPCR analysis (a, and b, left panel) or Northern blotting (b, right panel). For qPCR analyses in **a**-**b**, data are shown as mean ± SEM of three independent experiments. For Northern blotting analysis in **b**, two independent experiments were performed with similar results. ***, *P* < 0.001.

**Supplementary Fig. 11 GOLGA2P10 knockdown has no effect on the mRNA level of BCL-xL.** QGY-7703 cells were transfected with the indicated RNA duplexes, and then incubated with DMSO or tunicamycin for 12 hours, followed by qPCR analysis. iMAX, cells exposed to Lipofectamine RNAiMAX but not RNA duplexes. NC, negative control for siRNAs. β-actin was used as an internal control. Data are shown as mean ± SEM of three independent experiments. ns, not significant.

**Supplementary Fig. 12** **Effects of GOLGA2P10 knockdown on the expression of apoptosis regulatory genes.** QGY-7703 cells transfected with the indicated RNA duplexes were treated with tunicamycin for 12 hours before Western blotting. iMAX, cells exposed to Lipofectamine RNAiMAX but not RNA duplexes. NC, negative control for siRNAs. β-actin was used as an internal control. Two independent experiments were performed with similar results.

**Supplementary Fig. 13 Ectopic expression of GOLGA2P10 had no effect on the protein levels of MCL-1, BCL-2, BCL-w and BCL2A1.** SK-HEP-1 cells with stable overexpression of GOLGA2P10 (P10) or control (Ctrl) vector were treated with DMSO (-) or tunicamycin (+) for 6 hours, followed by Western blotting to detect the protein levels of MCL-1, BCL-2, BCL-w and BCL2A1. -, absence; +, presence. Two independent experiments were performed with similar results.

**Supplementary Fig. 14 GOLGA2P10 knockdown has no effect on the mRNA levels of UPR downstream genes.** QGY-7703 cells transfected with the indicated RNA duplexes were treated with DMSO or tunicamycin for 12 hours, and then subjected to qPCR analysis. iMAX, cells exposed to Lipofectamine RNAiMAX but not RNA duplexes. NC, negative control for siRNAs. β-actin was used as an internal control. Data are shown as mean ± SEM of three independent experiments. *, *P* < 0.05; **, *P* < 0.01; ***, *P* < 0.001; ns, not significant.

**Supplementary Fig. 15** **BAD knockdown antagonized the pro-apoptotic effect of GOLGA2P10 silencing.** **a** Knockdown effect of siRNA targeting BAD. SK-HEP-1 cells were transfected with the indicated RNA duplexes for 48 hours, and then subjected to Western blotting. **b** Knockdown effect of siRNA targeting BAX or BAK. QGY-7703 cells were transfected with the indicated RNA duplexes for 48 hours before qPCR analysis. **c** BAD knockdown antagonized the pro-apoptosis effect of GOLGA2P10 silencing. SK-HEP-1 cells were co-transfected with the indicated RNA duplexes, and then treated with DMSO or tunicamycin for 60 hours before DAPI staining. iMAX, cells exposed to Lipofectamine RNAiMAX but not RNA duplexes. NC, negative control for siRNAs. β-actin was used as an internal control. For Western blotting analysis in **a**, two independent experiments were performed with similar results. For **b**-**c**, data are shown as mean ± SEM of three independent experiments. **, *P* < 0.01, ***, *P* < 0.001.

**Supplementary Fig. 16**. **ER-stress or silencing GOLGA2P10 has no effect on the levels of GOLGA2 protein.** **a** Tunicamycin (Tm) or thapsigargin (Tg) treatment didn’t affect GOLGA2 level. QGY-7703 cells were treated with the Tm or Tg for 6 hours before Western blotting. DMSO, vehicle control for Tm and Tg. **b** Silencing GOLGA2P10 had no effect on GOLGA2 level. QGY-7703 cells were reversely transfected with the indicated RNA duplexes for 48 hours before Western blotting. iMAX, cells exposed to Lipofectamine RNAiMAX but not RNA duplexes. NC, negative control for siRNAs. siP10-1 and siP10-2, siRNAs targeting different regions of GOLGA2P10. Two independent experiments were performed with similar results.

**Supplementary Tables**

**Supplementary Table 1. Univariate and Multivariate Analysis of Factors**

**Associated with Recurrence-Free Survival**

| **Clinical Variables** | **Case Number** | | **HR (95% CI) ^a^** | ***P* ^a^** |
| --- | --- | --- | --- | --- |
| **Univariate analysis** |  |  | |  |
| GOLGA2P10 (High vs Low) **^b^** | 78/78 | 1.752 (1.175-2.631) | | **0.006** |
| Age (≤50 vs ＞5 yrs) | 84/72 | 0.847 (0.571-1.256) | | 0.409 |
| Gender (F vs M) | 21/135 | 0.678 (0.402-1.144) | | 0.145 |
| AFP (≤400 vs ＞400 ng/ml) | 80/76 | 1.269 (0.942-1.708) | | 0.117 |
| Tumor size (≤5 vs ＞5 cm) | 74/82 | 1.805 (1.209-2.697) | | **0.004** |
| Tumor number (1 vs >1) | 126/30 | 2.365 (1.498-3.734) | | **<0.0001** |
| HBV infection (- vs +)**^c^** | 30/126 | 1.263 (0.749-2.129) | | 0.381 |
| Differentiation (I/II vs III/IV) | 95/59 | 1.356 (0.907-2.027) | | 0.137 |
| Cirrhosis (- vs +)**^c^** | 18/138 | 1.324 (0.667-2.627) | | 0.423 |
| Child-Pugh (A vs B) | 152/4 | 1.807 (0.663-4.922) | | 0.247 |
| TNM (I vs II/III/IV) | 109/47 | 1.871 (1.238-2.828) | | **0.003** |
| **Multivariate analysis** |  |  | |  |
| TNM (I vs II/III/IV) | 109/47 | 1.984 (1.310-3.003) | | **0.001** |
| GOLGA2P10 (High vs Low) **^b^** | 78/78 | 1.847 (1.236-2.760) | | **0.003** |
| Abbreviations: AFP, α-fetoprotein; CI, confidence interval; HBV, hepatitis B virus; HR, hazard ratio.  **^a^** HR and *P* values were calculated using univariate or multivariate Cox proportional hazards regression.  **^b^** GOLGA2P10 level was examined in 156 HCC tissues by qPCR and normalized to β-actin level. The median value of the examined samples was chosen as the cut-off point to dichotomize patients into GOLGA2P10-low or GOLGA2P10-high groups.  **^c^** −, absence; +, presence. | | | | |

**Supplementary Table 2. Sequences of RNA and DNA oligonucleotides**

| **Name** | **Sense strand/Sense primer (5'-3')** | **Antisense strand/Antisense primer (5'-3')** |
| --- | --- | --- |
| **siRNA duplexes** | | |
| siPERK-1 | CCAGAGAAGUGGCAAGAAAdTdT | UUUCUUGCCACUUCUCUGGdTdT |
| siPERK-2 | GGAACGACCUGAAGCUAUAdTdT | UAUAGCUUCAGGUCGUUCCdTdT |
| siIRE1a | GGACGUGAGCGACAGAAUAdTdT | UAUUCUGUCGCUCACGUCCdTdT |
| siATF6 | GAACAGGAUUCCAGGAGAAdTdT | UUCUCCUGGAAUCCUGUUCdTdT |
| siATF4-1 | GGUGGCCAAGCACUUCAAAdTdT | UUUGAAGUGCUUGGCCACCdTdT |
| siATF4-2 | CACUCCAGAUCAUUCCUUUdTdT | AAAGGAAUGAUCUGGAGUGdTdT |
| siCHOP-1 | GGAAAUGAAGAGGAAGAAUdTdT | AUUCUUCCUCUUCAUUUCCdTdT |
| siCHOP-2 | UGAUUGACCGAAUGGUGAAdTdT | UUCACCAUUCGGUCAAUCAdTdT |
| siP10-1 | CCAUCAGUGUGUUCUGGUUdTdT | AACCAGAACACACUGAUGGdTdT |
| siP10-2 | GGGUCUGAGAGUGGGCAAdTdT | UUGCCCACUCUCAGACCCdCdG |
| siBAX-1 | GAACUGAUCAGAACCAUCAdTdT | UGAUGGUUCUGAUCAGUUCdTdT |
| siBAX-2 | GACCAGGGUGGUUGGGUGAdTdT | UCACCCAACCACCCUGGUCdTdT |
| siBAK-1 | CGUGCUGGUGGUUCUGGGUdTdT | ACCCAGAACCACCAGCACGdTdT |
| siBAK-2 | CAGAGAAUGCCUAUGAGUAdTdT | UACUCAUAGGCAUUCUCUGdTdT |
| siBCL-xL-1 | GCUUUGAACAGGUAGUGAAdTdT | UUCACUACCUGUUCAAAGCdTdT |
| **Supplementary Table 2. Sequences of RNA and DNA oligonucleotides (continued)** | | |
| **Name** | **Sense strand/Sense primer (5'-3')** | **Antisense strand/Antisense primer (5'-3')** |
| **siRNA duplexes** | | |
| siBCL-xL-2 | GGAUGGCCACUUACCUGAAdTdT | UUCAGGUAAGUGGCCAUCCdTdT |
| NC | UUGUACUACACAAAAGUACUG | GUACUUUUGUGUAGUACAGUU |
| **Primers for cloning (restriction enzyme sites are underlined; 5'-3')** | | |
| del-ATG-GFP | TCACTCGAGGTGAGCAAGGGCGAGGAGCT | TCAGGGCCCTGGACGAGCTGTACAAGTAA |
| GAPDH-GFP | TCAGAATTCCTGTTTCATCCAAGCGTGTAA | TCACTCGAGGTTAAAAGCAGCCCTGGTGA |
| ORF | ACAAAGCTTAGGCAGCCTATTGTCTTTCT | ATGGGATCCGCCCGGCCTGGGTGGCAG |
| P (−1222/+175) | GTACTCGAGGGTTGTGGGGGTATATTCCA | TCAAAGCTTCGACCCCTCCTCAAATCACA |
| P (−435/+175) | GTACTCGAGCACGAGCACACAGAGAAAGG | TCAAAGCTTCGACCCCTCCTCAAATCACA |
| P(−201/+175) | GTACTCGAGCTTGCTCCGCGTGTACTCA | TCAAAGCTTCGACCCCTCCTCAAATCACA |
| P(−125/+175) | GTACTCGAGTTGTAGCCGGTGCGCGGGGT | TCAAAGCTTCGACCCCTCCTCAAATCACA |
| P(∆C) | AGGACTCGCGCCCTCTTGCGTGCCCC | CAAGAGGGCGCGAGTCCTCCCGTGAGT |
| GOLGA2P10 | GAGGAATTCAGGCAGCCTATTGTCTTTCTC | TGCAAGCTTCCCAGAGGGCAGGTAAATGT |
| BCL-xL | ATAGCTAGCGCCACCATGTCTCAGAGCAACCGGGA | ATAGAATTCTCATTTCCGACTGAAGAGTGAG |
| gab-BAD | ATAAAGCTTGCCACCATGTTCCAGATCCCAGAGTTT | ATAGAATTCTCACTGGGAGGGGGCGGA |
| gab-S75A-BAD | CGCCACAGCGCCTACCCCGCGGGGACGG | GCGGGGTAGGCGCTGTGGCGACTCCGGATC |
| **Supplementary Table 2. Sequences of RNA and DNA oligonucleotides (continued)** | | |
| **Name** | **Sense strand/Sense primer (5'-3')** | **Antisense strand/Antisense primer (5'-3')** |
| **Primers for qPCR** | | |
| β-actin | CTCGCCTTTGCCGATCC | GTGAGGATGCCTCTCTTGCTC |
| AK124097 | CCCTTCAGCACTCTACGTCA | TCTTTTGAGGGACTGTCCGA |
| AX800134 | TGATGTTGAACCAATGCACCA | ATAGCTTTGCGAAAAATGGACACA |
| BC070200 | TCTCAAGCGGCAGAGCAGTT | TCTCCAAAGGCCCAAAGAGAC |
| MIR4435-2HG | GCATGAGTCATCTCGTTCCAA | GAGCTTCCTGTTTCATCTCCCA |
| GOLGA2P10 | CCTTGCTGGGCCCTTCTG | CTTTTCCTCTGGACCTCGGT |
| MALAT1 | TCGTTTGCCTCAGACAGGTA | GGAAGGGGTCAGGAGAAAGTG |
| PERK | CTCCCACCTCAGCGACG | CGGTCGCAACTCTGTCTCAT |
| IRE1a | CACCTCCACTCCCTCAACAT | ACAGTCTTCGCTCAGCATCT |
| ATF6 | CAGGAACTCAGGGAGTGAGC | CCTGGTGTCCATCACCTGAC |
| ATF4 | GTCCCTCCAACAACAGCAAG | ACTTTCTGGGAGATGGCCAA |
| CHOP | TGGAAGCCTGGTATGAGGAC | TGTGACCTCTGCTGGTTCTG |
| GRP78 | CGAGGAGGAGGACAAGAAGG | CACCTTGAACGGCAAGAACT |
| GRP94 | GGATGGTCTGGCAACATGGA | ATACCCTGACCGAAGCGTTG |
| DR5 | ACCCAACAAGACCTAGCTCC | CTGTGTTTCTGGTCGTGGTG |
| **Supplementary Table 2. Sequences of RNA and DNA oligonucleotides (continued)** | | |
| **Name** | **Sense strand/Sense primer (5'-3')** | **Antisense strand/Antisense primer (5'-3')** |
| GADD34 | CCTCTGGCAATCCCCCATAC | CAGCCCCAGTGTTTCTCTGT |
| BCL-xL | CCCAGGGACAGCATATCAGA | GAAGAGTGAGCCCAGCAGAA |
| BAX | GTCGCCCTTTTCTACTTTGCC | GTGAGGAGGCTTGAGGAGTC |
| BAK | GGCTGCACAGGGACAAGTAA | TCAGTGGAGGACGGGATCAG |
| **Primers for ChIP** | | |
| GOLGA2P10 | GCCCAGGTGCCTTACTATTG | TCTGCGAGTCCTCCCGT |
| DR5 | AAGGAGAGAACAGAAGGGGC | TGAGGTTCAGAGTCCGCTTT |
| GAPDH | AAAAGCGGGGAGAAAGTAGG | AAGAAGATGCGGCTGACTGT |
| **Probes for Northern Blotting** | | |
| GOLGA2P10 | TCCCACCTGCCGCCTTCTGTCCAGCACTTGCATGACACTTCCCTCT | |
| β-actin | GGGATGCTCGCTCCAACCGACTGCTGTCACCTTCACCGTTCCAGTTTT | |
| **Probes for EMSA** | | |
| GOLGA2P10-probe | GACTCGCAGACGTTACTGCCCTCTTGCGTGCCCCG | CGGGGCACGCAAGAGGGCAGTAACGTCTGCGAGTC |
| CHOP consensus | CGAGGGGGGATTGCATCTGGAT | ATCCAGATGCAATCCCCCCTCG |
| non-specific oligo | GACCGCCGTGCCCACTGCTCGCCTCGGTTGCC | GGCAACCGAGGCGAGCAGTGGGCACGGCGGTC |

**Supplementary Table 3. Amplification efficiency of qPCR primers and Tm of the amplicon calculated by the dissociation curve**

| **Primers for qPCR** | **Amplification efficiency** | **Tm (℃)** |
| --- | --- | --- |
|  |  |  |
| β-actin | 1.873 | 82 |
| AK124097 | 2.062 | 79 |
| AX800134 | 1.936 | 80 |
| BC070200 | 1.821 | 85 |
| MIR4435-2HG | 2.010 | 83 |
| GOLGA2P10 | 2.035 | 84 |
| MALAT1 | 1.918 | 80 |
| PERK | 1.855 | 89 |
| IRE1a | 1.921 | 85 |
| ATF6 | 1.879 | 81 |
| ATF4 | 1.943 | 83 |
| CHOP | 1.861 | 83 |
| GRP78 | 2.024 | 87 |
| GRP94 | 1.880 | 81 |
| DR5 | 1.987 | 83 |
| GADD34 | 1.966 | 83 |
| BCL-xL | 2.27 | 86 |
| BAX | 1.820 | 85 |
| BAK | 1.866 | 87 |
| ChIP-GOLGA2P10 | 1.873 | 85 |
| ChIP-DR5 | 2.130 | 82 |
| ChIP-GAPDH | 2.012 | 89 |
