# Supplementary figures and images for: LncRNA GOLGA2P10 is induced by PERK/ATF4/CHOP signaling and protects tumor cells from ER stress-induced apoptosis by regulating Bcl-2 family members
Source: Cell Death Dis. 2020 Apr 24;11(4):276. doi: 10.1038/s41419-020-2469-1 (PMC7181651; doi:10.1038/s41419-020-2469-1)

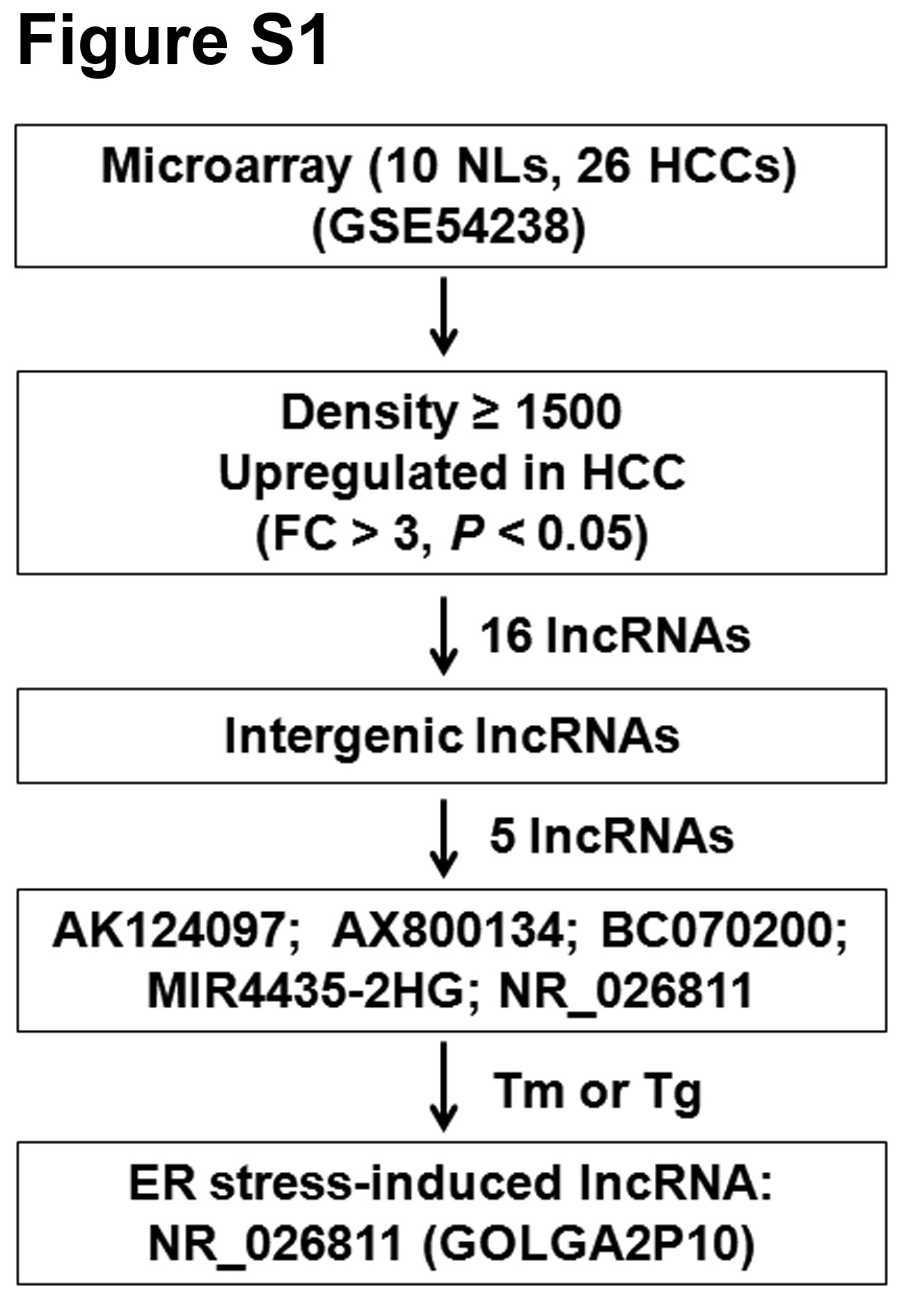

Supplement: Supplementary file 2 — Figure S1 [file 41419_2020_2469_MOESM2_ESM.tif]

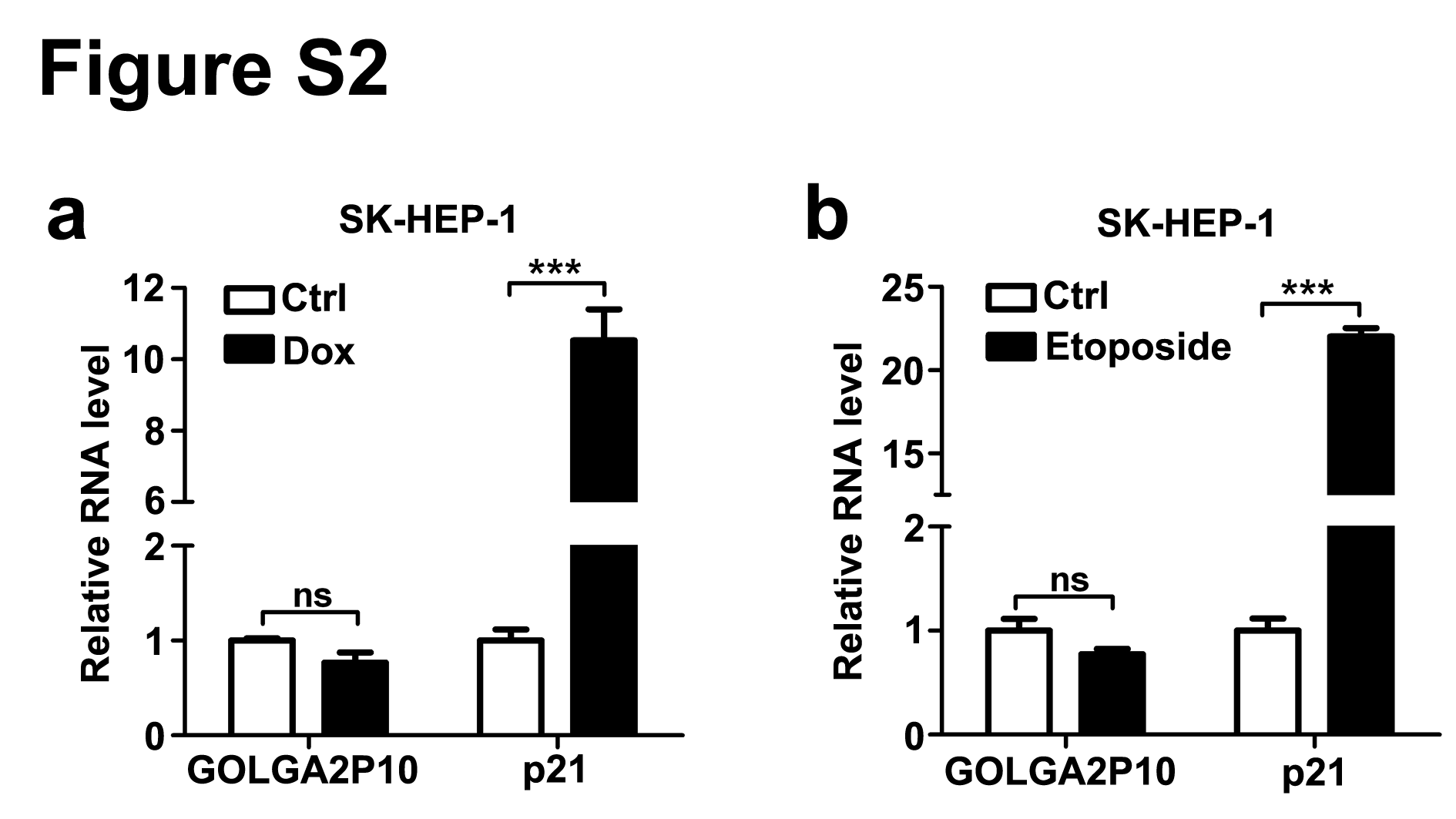

Supplement: Supplementary file 3 — Figure S2 [file 41419_2020_2469_MOESM3_ESM.tif]

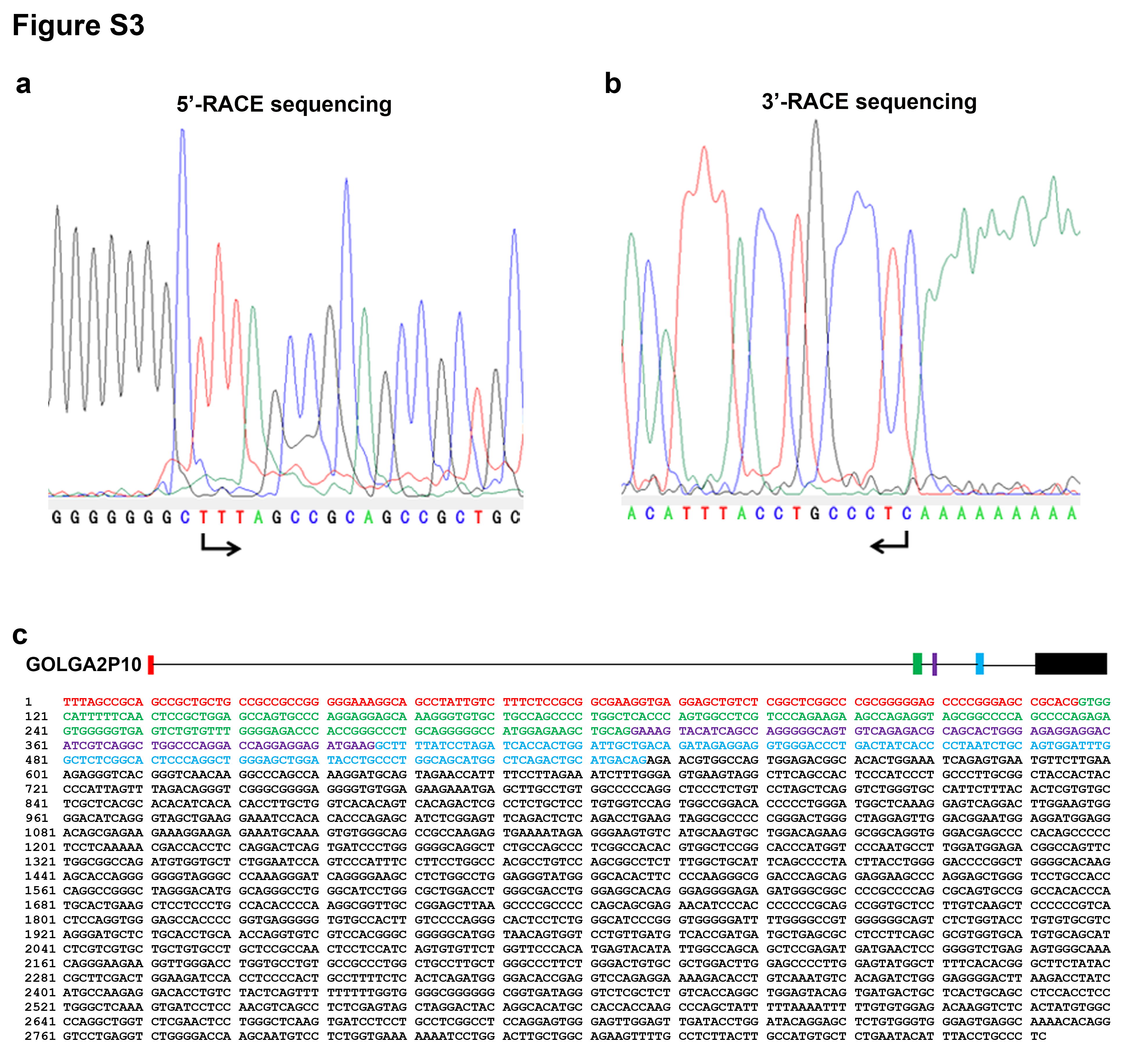

Supplement: Supplementary file 4 — Figure S3 [file 41419_2020_2469_MOESM4_ESM.tif]

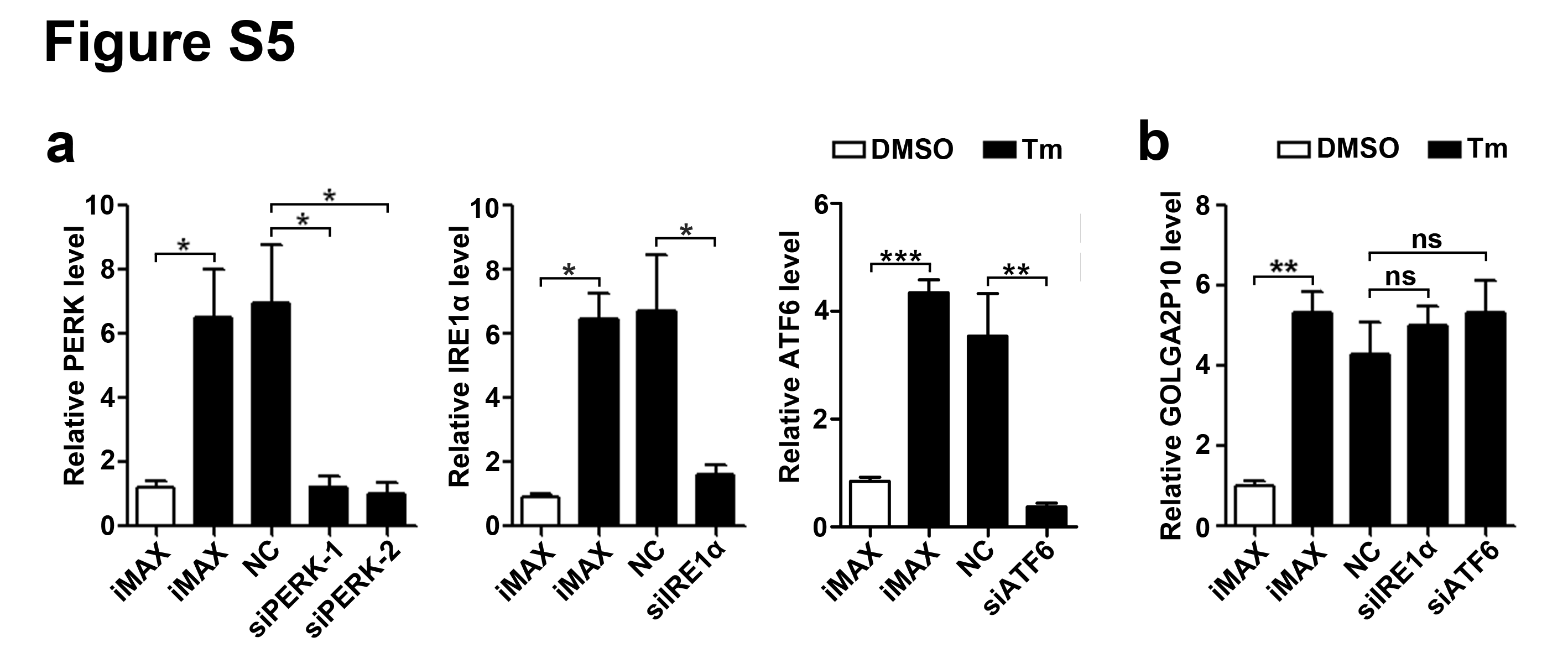

Supplement: Supplementary file 5 — Figure S4 [file 41419_2020_2469_MOESM5_ESM.tif]

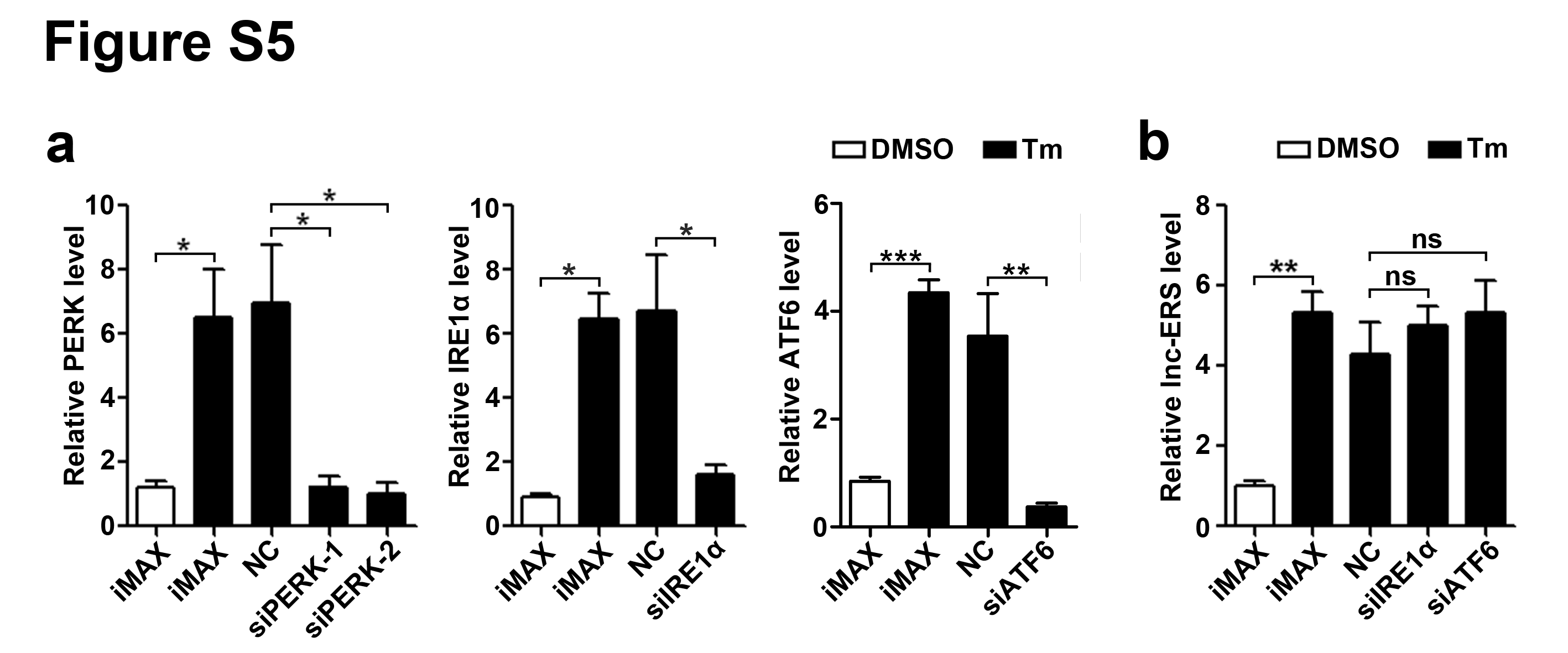

Supplement: Supplementary file 6 — Figure S5 [file 41419_2020_2469_MOESM6_ESM.tif]

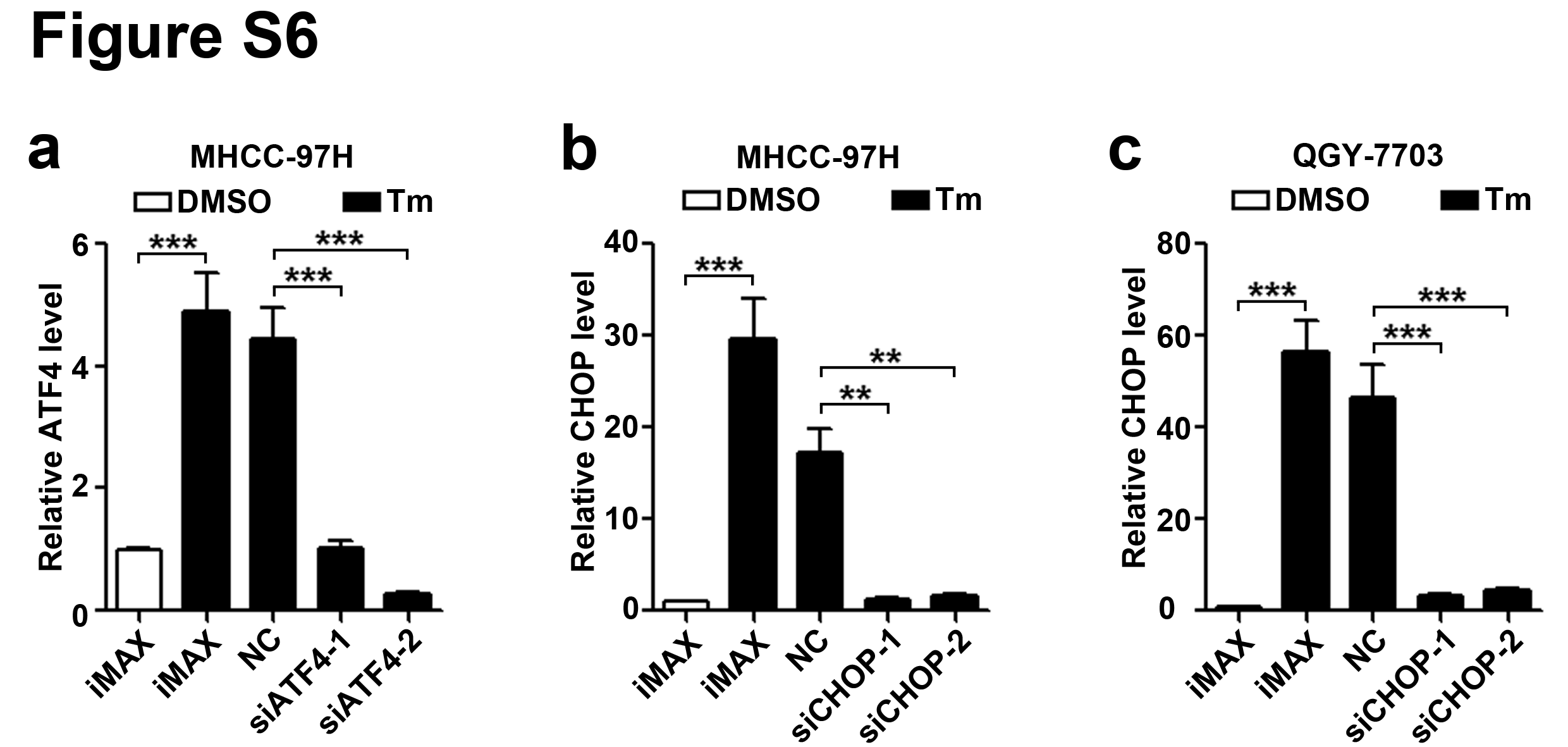

Supplement: Supplementary file 7 — Figure S6 [file 41419_2020_2469_MOESM7_ESM.tif]

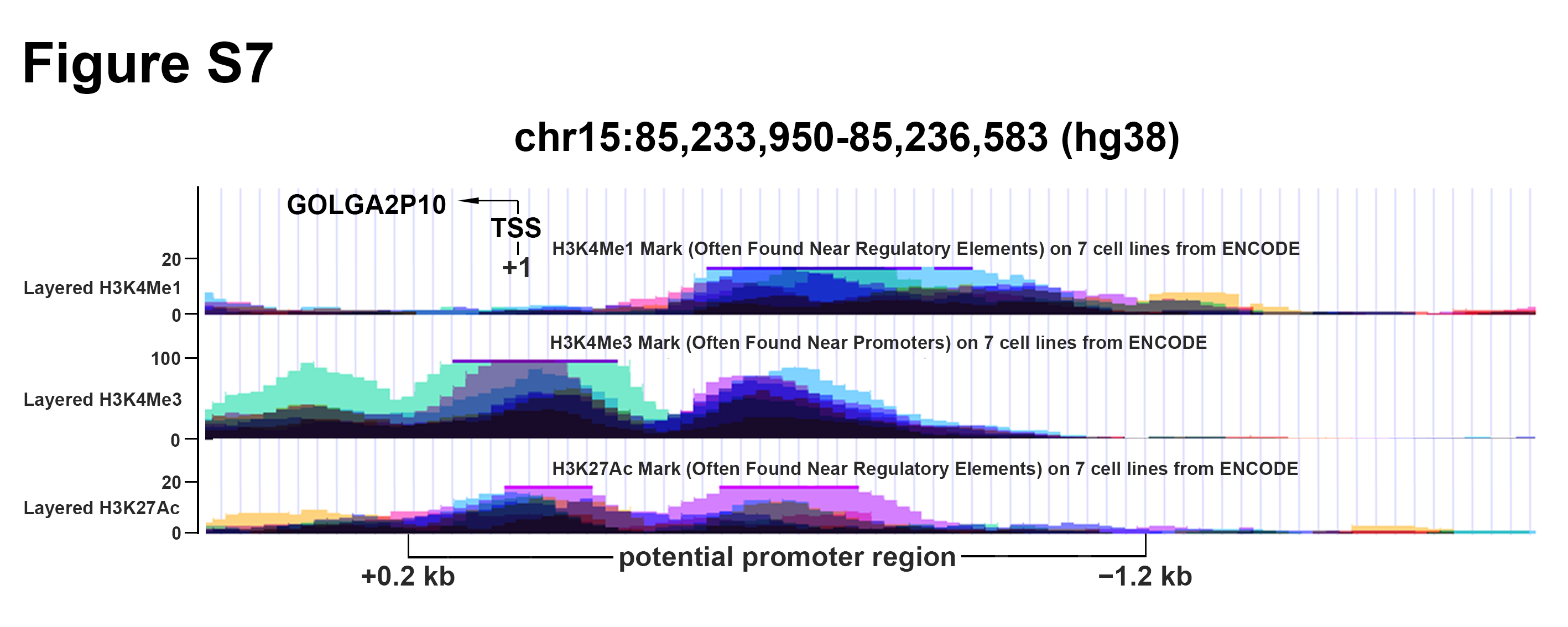

Supplement: Supplementary file 8 — Figure S7 [file 41419_2020_2469_MOESM8_ESM.tif]

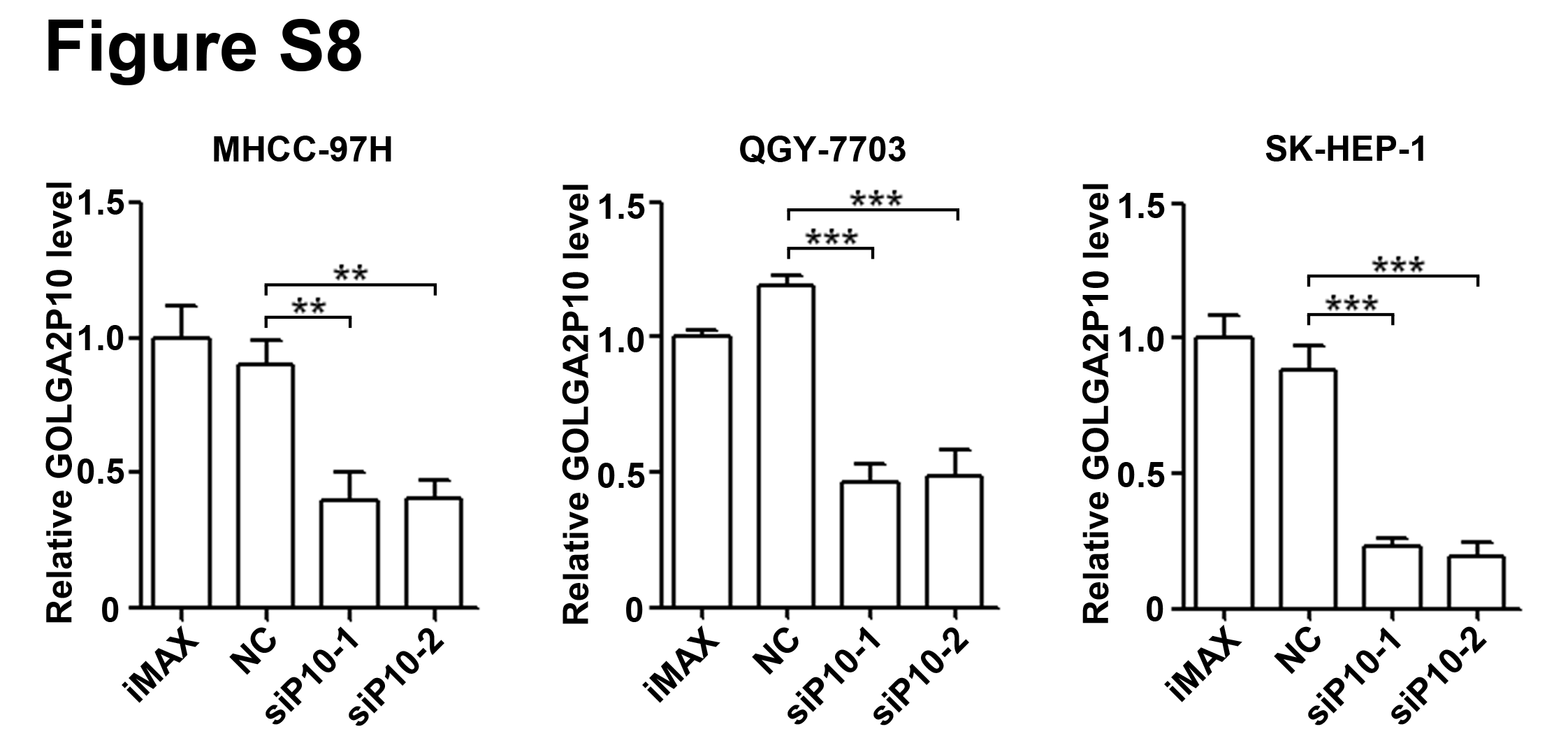

Supplement: Supplementary file 9 — Figure S8 [file 41419_2020_2469_MOESM9_ESM.tif]

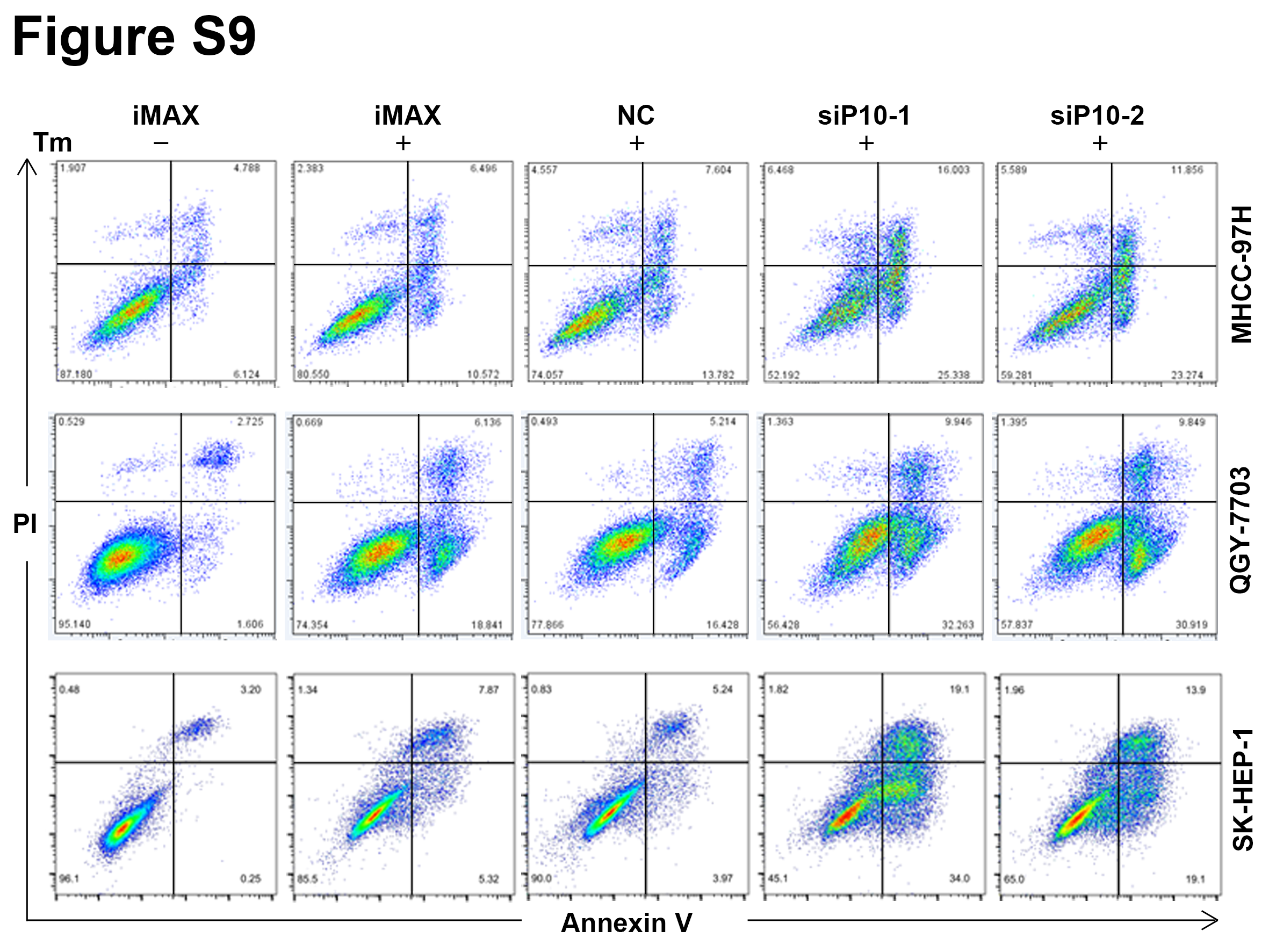

Supplement: Supplementary file 10 — Figure S9 [file 41419_2020_2469_MOESM10_ESM.tif]

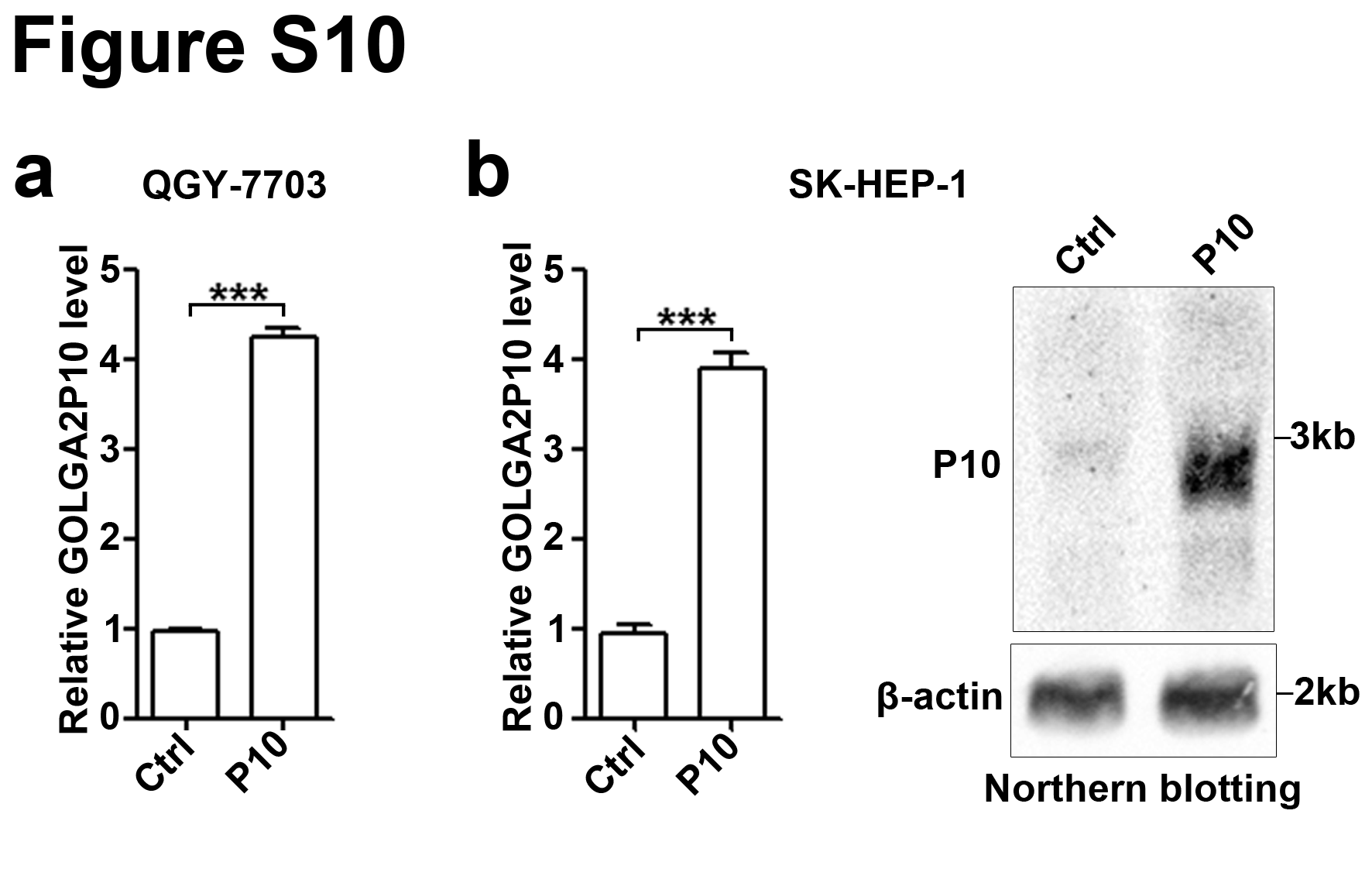

Supplement: Supplementary file 11 — Figure S10 [file 41419_2020_2469_MOESM11_ESM.tif]

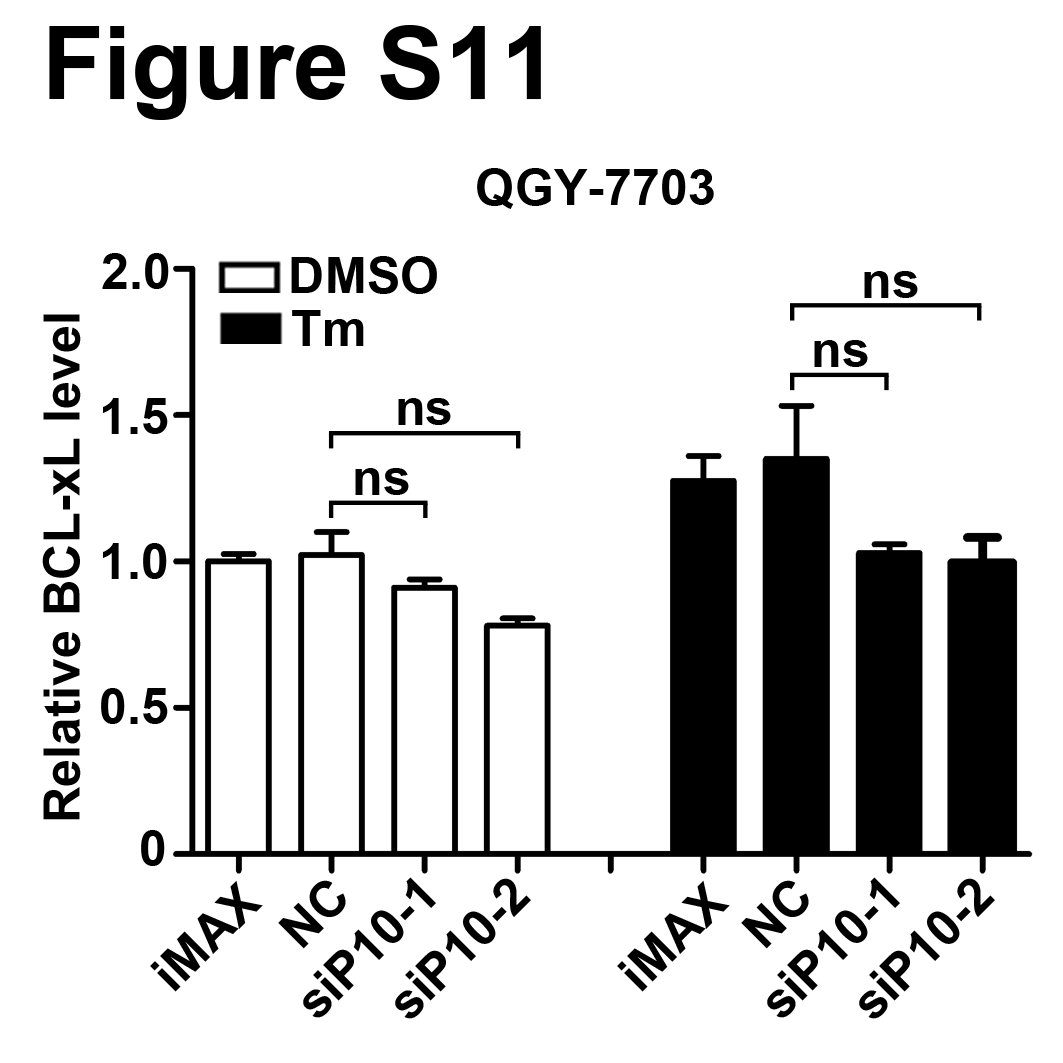

Supplement: Supplementary file 12 — Figure S11 [file 41419_2020_2469_MOESM12_ESM.tif]

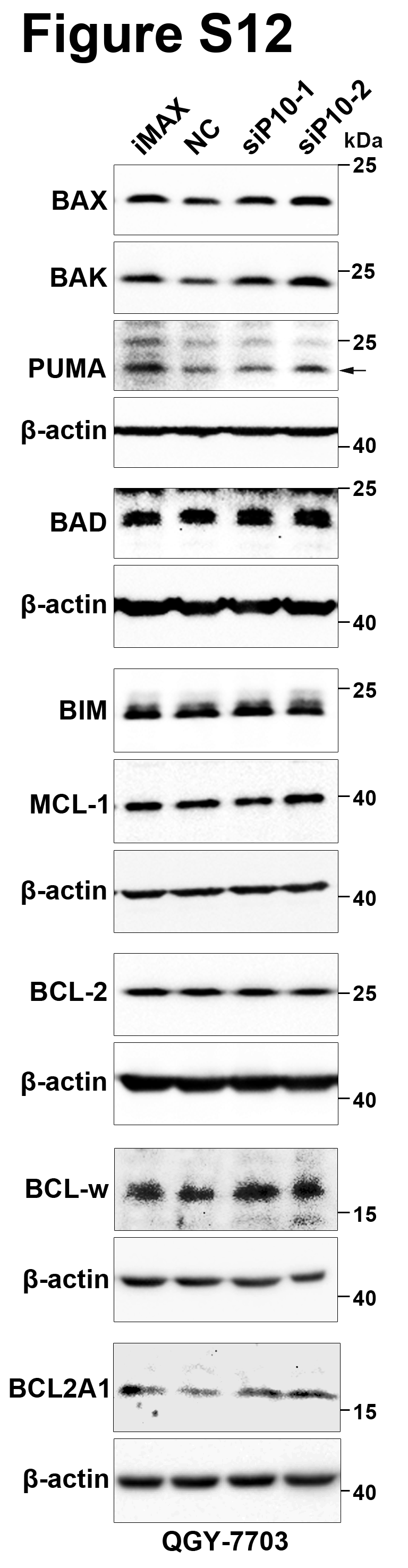

Supplement: Supplementary file 13 — Figure S12 [file 41419_2020_2469_MOESM13_ESM.tif]

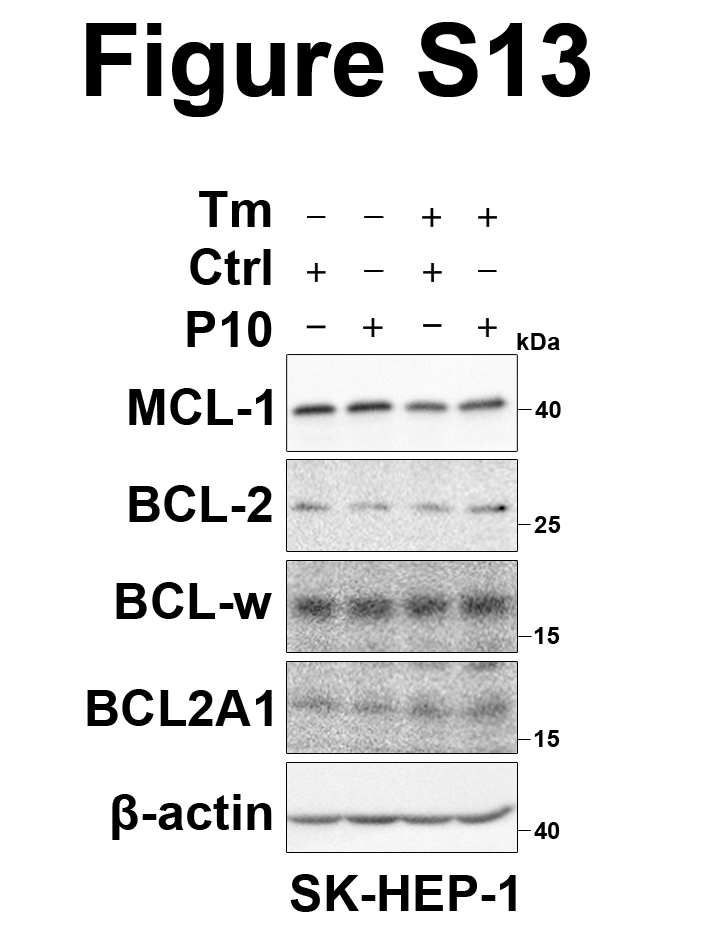

Supplement: Supplementary file 14 — Figure S13 [file 41419_2020_2469_MOESM14_ESM.tif]

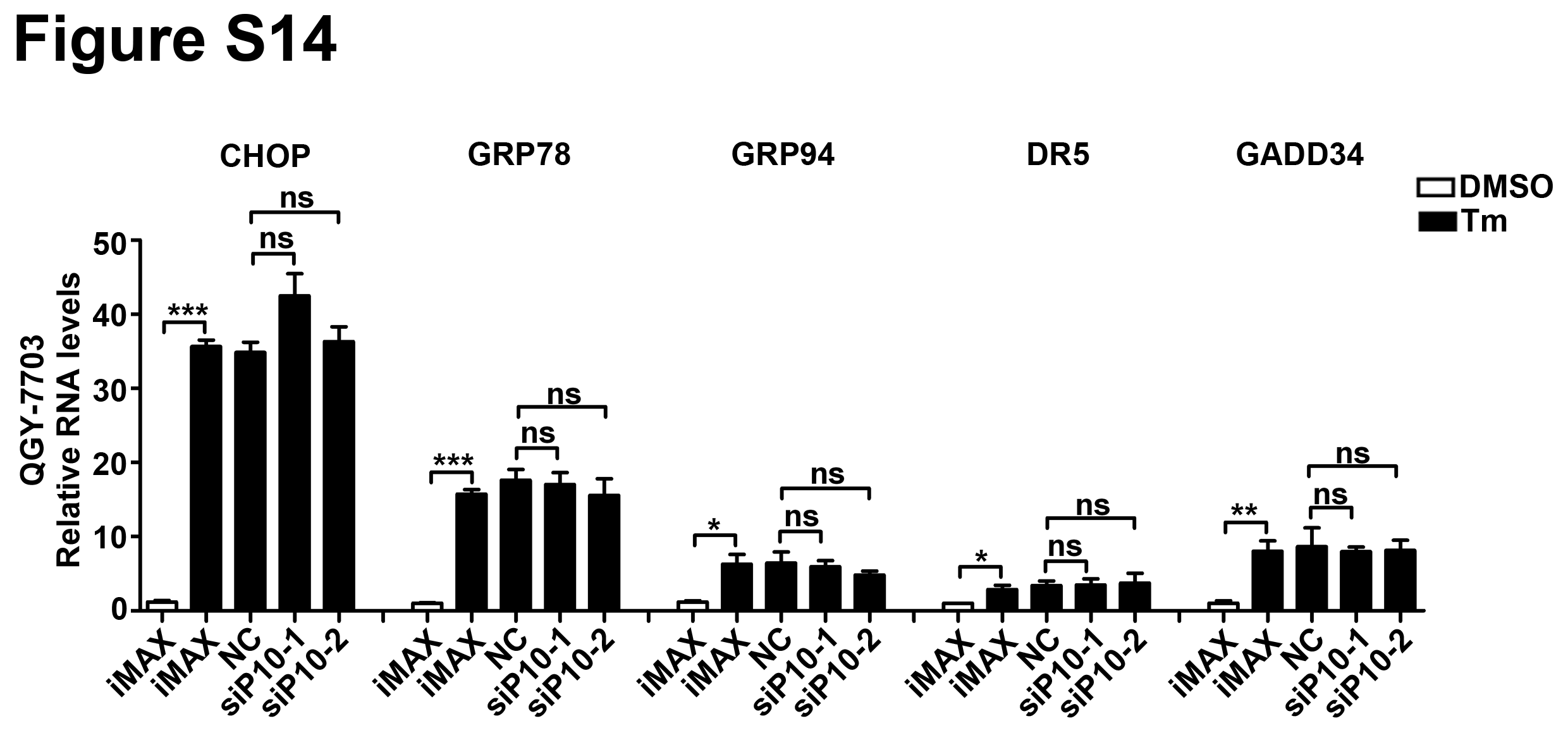

Supplement: Supplementary file 15 — Figure S14 [file 41419_2020_2469_MOESM15_ESM.tif]

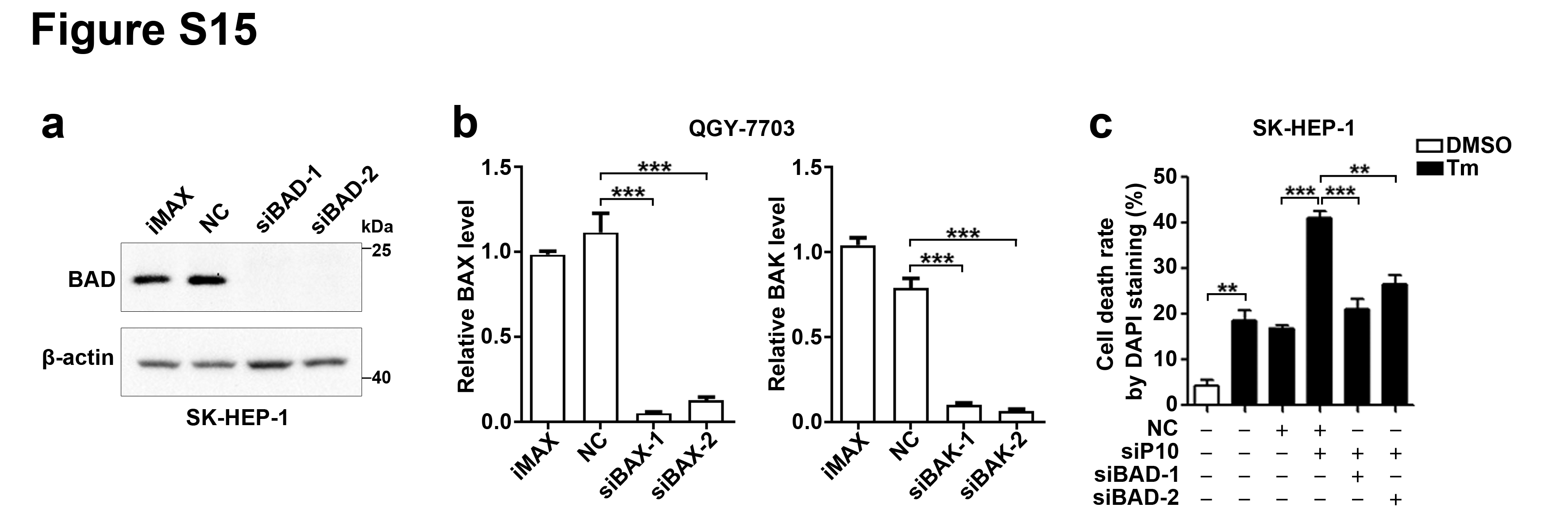

Supplement: Supplementary file 16 — Figure S15 [file 41419_2020_2469_MOESM16_ESM.tif]

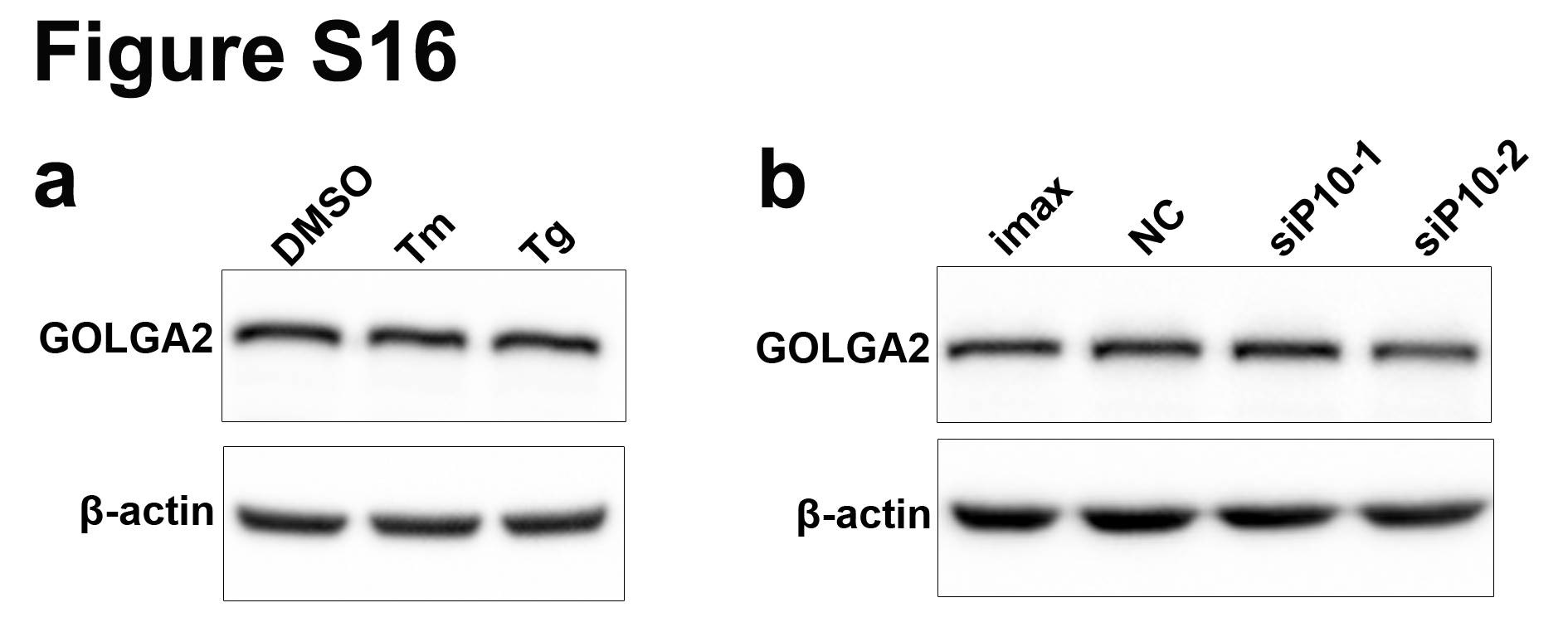

Supplement: Supplementary file 17 — Figure S16 [file 41419_2020_2469_MOESM17_ESM.tif]
